# Optimisation of selection for methane mitigation by integrating production traits with ruminal microbiome-driven breeding in beef cattle

**DOI:** 10.1186/s12711-026-01053-w

**Published:** 2026-07-09

**Authors:** Tuan Q. Nguyen, Marina Martínez-Álvaro, Joana Lima, Gregor Gorjanc, Matthew A. Cleveland, Rainer Roehe

**Affiliations:** 1 Scotland’s Rural College, Peter Wilson Building, The King’s Buildings, West Mains Road, Edinburgh, EH9 3JG UK; 2 Institute of Animal Science and Technology, Universitat Politècnica de Valéncia, 46010 Valencia, Spain; 3 The Roslin Institute, University of Edinburgh, Easter Bush, Edinburgh, EH25 9RG UK; 4 Genus Plc, DeForest, WI 53532 USA

## Abstract

**Background:**

Breeding for reduced methane production is challenged by the high cost associated with its continuous measurements on individual animals. To address this, a microbiome-driven breeding methodology was developed and investigated to identify the most informative microbial genes (MGs) related to methane production. The analyses considered different genomic selection scenarios based on data from beef cattle: (i) with and without inclusion of production traits such as daily feed intake, average daily gain (ADG), cold carcass weight (CCW); (ii) different sets of MGs were evaluated - those most informative to predict the host genetics of methane production, with and without restriction of showing favourable genetic correlation with ADG.

**Results:**

Methane production had a high heritability (0.56), and we identified 43 MGs, whose abundances are associated with methane production through functions such as electron transfer and coenzyme A biosynthesis. Selection of 10% of the animals based on microbiome-driven breeding using these 43 MGs revealed a methane mitigation potential of 18% per generation, slightly higher than 17% reduction achieved through direct selection based on methane measurements using respiration chamber. Integrating production traits into the microbiome-driven breeding system enhanced the methane reduction to 23% but also reduced ADG by 14%. To avoid the decline in ADG and CCW, residual methane production was developed on genetic level, which yielded a meaningful 15% reduction in methane production, and a correlated improvement in residual feed intake of 5%. Applying microbiome-driven breeding based only on profiles of MGs favourably correlated with both methane production and ADG resulted in a promising response in methane mitigation of 14%.

**Conclusions:**

The high heritability of methane production supports animal breeding programmes aimed at reducing methane production. However, selection solely for lower methane production resulted in substantial adverse responses in key production traits. Therefore, it is essential to adopt specific selection criteria, such as residual methane production, to reduce its emissions while avoiding negative correlated responses in production traits. Especially, microbiome-driven breeding based on MGs favourably correlated with both reduced methane production and improved ADG, is recommended to directly enhance the efficiency of ruminal microbial metabolism to achieve sustainable beef production by cost-effective breeding.

**Supplementary Information:**

The online version contains supplementary material available at 10.1186/s12711-026-01053-w.

## Background

Methane (CH4) is a highly potent greenhouse gas that significantly contributes to increasing global temperatures and climate change. Due to its simple molecular structure and ability to absorb infrared radiation, CH4 can trap heat 27–30 times more effectively than CO_2_ over a 100-year period [1–3]. Globally, the agrifood sector has produced 5.28 gigatonnes of CO_2_ equivalent from CH4 [4], with enteric fermentation from livestock accounting for 78% of total emissions. As global livestock production may expand to meet the growing demand for animal protein of an increasing world population, there is a risk of increased CH4 emissions from ruminant livestock. Therefore, advancements in reducing CH4 emissions from ruminants represent a crucial contribution to mitigating global warming and its impacts on climate change.

In addition to contributing to climate change, CH4 emissions from ruminants may be directly linked to productivity losses, as up to 12% of feed energy can be lost through CH4 metabolism [5]. However, ruminants have a unique capability to convert fibrous feeds such as grass, which originate from extensive grasslands unsuitable for arable production and are largely indigestible by monogastric animals, into high-quality meat and milk. Ruminal fermentation is a complex process carried out by microorganisms such as bacteria, fungi, and protozoa, collectively referred to as microbiota. The microbiota hydrolyses complex carbohydrates into monosaccharides, which are then further fermented to produce volatile fatty acids (VFAs), mainly acetate, propionate, and butyrate [6]. These VFAs are absorbed and serve as the main energy sources for the host animal. During fermentation, excess molecular hydrogen (H_2_), along with others, such as formic acid and carbon dioxide, can be produced, which can affect the ruminal metabolism through negative feedback mechanisms [7, 8]. To prevent this inhibition, methanogenic archaea in the rumen use H_2_ to reduce CO_2_ and produce CH4 through methanogenesis: CO_2_ + 4H_2_ → CH4 + 2H_2_O. This process helps maintain a low partial pressure of H_2_, allowing fermentation to proceed efficiently. Consequently, CH4 production (CH4P) is strongly linked to excess hydrogen metabolism during fermentation and is dependent on factors such as animal genetics, feed intake, dietary composition and feed additives.

A large body of research has been published on mitigation strategies targeting CH4P through management practices, including diet formulation, chemical inhibitors, fermentation modifiers, vaccines or early life programming [9]. These authors reviewed the strategies and concluded that they were likely to produce small to moderate reductions in CH4P, with the possible exception of chemical inhibitors, which could result in long-term 20–40% reductions in commercial farms. However, discussions persist regarding the limited understanding of these inhibitors in relation to consumer acceptance, safety, cost-effectiveness and product quality as well as animal health and reproduction.

Animal breeding has been proposed as a potential solution, however, there are several challenges such as the costly measurement of CH4P continuously from large numbers of individual animals and the lack of knowledge about the genetic correlations between CH4P and economically important production traits [9–11]. To the best of our knowledge, there are no studies on the impact of selection for mitigation of CH4P on the correlated selection responses of production traits in beef cattle, such as feed efficiency, growth rate, and a carcass characteristic.

Another area of research involves the use of ruminal microbiome profiles to identify animals inheriting lower CH4P. Roehe et al. [12] demonstrated the influence of host genetics on the ruminal microbial community structure, defined as the ratio of archaea to bacteria to be correlated with CH4 emissions. They also identified the high informativeness of the microbial genes (MGs) annotated in the Kyoto Encyclopedia of Genes and Genomes (KEGG) to predict CH4 emissions. Building on this, Martínez-Álvaro et al. [13] provided a comprehensive evaluation of the host heritabilities and genetic correlations between CH4 yield, a ratio trait between daily CH4P (g/day) and daily dry matter intake (kg/day) (CH4Y), and the abundance of various characteristics of the rumen microbiome, including functional KEGG MGs, microbial genera, and metagenome-assembled genomes. The authors developed microbiome-driven breeding (MDB), a genetic evaluation system based on a multiple-trait model including the abundances of the most informative MGs, to reduce CH4Y. Microbiome-driven breeding showed, depending on the selection intensity, a substantial mitigation potential of up to 17% of the mean CH4Y per generation, which was even higher than that of 13% achieved based on direct measurement of CH4Y using respiration chambers. These findings from Martínez-Álvaro et al. [13] have significant implications for breeding, as collecting rumen samples and performing metagenomic analyses are arguably more cost-effective than measuring CH4Y in a respiration chamber and other potential measurement techniques [14]. Furthermore, the stability of rumen microbiome profiles over time and their consistent phenotypic and genetic associations with CH4 traits indicate that one sample per animal is sufficient [15]. To reduce the generation interval, this sample should be collected at the beginning of the finishing phase in beef cattle. In the study of Martínez-Álvaro et al. [13], CH4Y was used as the target trait. There have been concerns regarding the heritability of the ratio trait CH4Y, particularly the possibility that it can result into non-zero heritability due to the genetics of feed intake, even when the heritability of CH4P is zero [10, 16]. In addition, selection for the ratio trait can produce unpredictable outcomes because there is no clear direction of how selection pressure will be affecting each of the traits in the ratio [17, 18]. Therefore, the present study explored the application of MDB using daily CH4P and its impact on other production traits.

The integration of production traits within MDB is even more important when daily CH4P rather than CH4Y is targeted, as it is expected to be highly correlated with feed intake [11, 19–21]. One possible breeding strategy of breeding could be based on an index combining production traits and CH4P. However, determining the weighting of these traits in the index presents a major challenge. To avoid this, one strategy is to estimate residual CH4P (RCH4) that is independent of production traits. This residual trait offers several advantages, including defining the direction of selection, avoiding adverse effects on genetically correlated traits and facilitating the monitoring of selection outcomes. Kennedy et al. [22] emphasised that residual traits should be genetically independent of the traits used in the model of their estimation. Therefore, the aim of this study was to derive RCH4 at the genetic level based on genomic breeding values (GEBVs) estimated using a multiple-trait model. The same approach was used to derive residual feed intake (RFI), the feed efficiency trait of this study. Tempelman and Lu [23] recommend RFI as the most relevant trait for improving metabolic efficiency.

As the basis for various selection scenarios to optimise breeding for CH4 mitigation, we estimated the heritabilities and genomic correlations between CH4P and key production traits in beef cattle, including daily feed intake (DFI), average daily gain (ADG), and cold carcass weight (CCW), using a Bayesian genomic analysis. First, we compared the expected accuracies and selection responses of genomic selection based on measured CH4P with those of various MDB strategies, incorporating the most informative MGs with and without the constraint of being favourably genetically correlated with ADG. Second, we integrated production traits and MDB into multivariate analyses to assess their impact on the accuracy of CH4P estimation and the resulting selection response. Third, we evaluated the impact of selection for measured CH4P on production traits using the estimated parameters and within a sensitivity analysis assuming substantially lower genetic correlations equal to the estimated phenotypic correlations. In addition, we developed RFI and RCH4 at the genetic level, aiming to reduce CH4P without compromising production traits of beef cattle and explored how selection on each of these traits influences the others.

## Methods

### Animals

Data for this study were collected from 359 beef steers from various experiments spanning five years (2011 (70), 2012 (72), 2013 (70), 2014 (71), and 2017 (76)), all conducted under uniform farm conditions at Scotland’s Rural College (SRUC) Beef and Sheep Research Centre. The experiments were approved by SRUC's Animal Experiment Committee under the UK Animals (Scientific Procedures) Act 1986. The cattle were from different breeds, including Aberdeen Angus-Limousin rotational crosses, Charolais-crosses, and purebred Luing and were fed two basal diets with forage-to-concentrate ratios of 480:520 g and 80:920 g (dry matter basis). Two breeds and two diets were primarily compared in each experiment, with an equal number of animals per treatment at the start of the trial. To optimise the experimental design, a power analysis for the breed, diet and sire effects was carried to determine the optimal number of sires and progeny per sire, ensuring sufficient statistical power in each experiment to detect potential differences in breed, diet and sire effects on CH4 emissions. The observed power, calculated using the estimates of sire and diet effects from the first trial of the present dataset and reported in Roehe et al. [12], was 85%. DFI (kg/day) was based on recorded individually feed intake by electronic feeders multiplied by the dry matter content of the diet. Body weight was measured weekly during each experiment and regressed on the 56 days of the performance test period to obtain ADG (kg/day). The CCW (kg) and the animal’s age were recorded at slaughter. Due to missing values, only 332 and 352 records were available for DFI and ADG, respectively, so that the feed conversion ratio (FCR) could only be calculated based on the lower number of observations. The FCR was not studied further but could be analysed, even on those animals with one component missing, using a Bayesian multiple-trait analysis as suggested by Shirali et al. [24] and Islam et al. [25].

### Methane emission data

After the performance test, groups of six animals were moved each week into individual training pens, which were identical in size and shape to the respiration chambers, for a one-week acclimation period. The following week, the group moved into six indirect open-circuit respiration chambers, where each animal remained for 72 h. The last 48 h were used to measure CH4P, which was calculated as grams of CH4 produced per day. Detailed descriptions of the respiration chambers and measurement techniques is available in Rooke et al. [26] and of the experiments in Troy et al. [27], Roehe et al. [12], and Duthie et al. [28]. Since not all performance tested animals could be measured for CH4P, e.g., in the 2017 experiment, only a sample of 12 out of 76 animals were measured, so that only 287 cattle had daily CH4P records.

### Host DNA samples

Blood samples were collected from 359 beef cattle for DNA collection, either from the jugular or coccygeal vein, in live animals or during slaughter. Blood samples were placed in tubes with 1.8 mg EDTA/ml and stored at −20 °C. Additional blood or semen samples were collected from siblings and sires of the cattle totalling 393 samples for host DNA extraction. Genomic DNA was isolated from blood using the Qiagen QIAamp toolkit, and from semen the Qiagen QIAamp DNA Mini Kit, following the manufacturer's instructions. The DNA concentration and quality were evaluated using a Nanodrop ND-1000. The genotyping process was performed by Neogen Genomics with the GeneSeek Genomic Profiler (GGP) BovineSNP50k Chip. The obtained genotypes were filtered through quality control measures in PLINK version 1.09b [29], which are described in detail in Martínez-Álvaro et al. [30]. After quality control, 385 animals and 36,780 SNPs were used for further genomic analyses.

### Collection of rumen samples, metagenomic sequencing of samples and data transformation

At slaughter, postmortem rumen digesta samples (approximately 50 ml) from the 359 steers were obtained as soon as the rumen was opened. The ruminal fluid was filtered to remove solid undigested materials. Then, five millilitres of filtered fluid were mixed with ten millilitres of phosphate-buffered saline containing 87% glycerol and stored at −20 °C. DNA extraction from rumen samples was performed using the Yu and Morrison 2004 methodology [31], and DNA concentrations and integrity were assessed by Nanodrop ND-1000. Samples from four animals were removed due to insufficient quality. Genomic DNA libraries were prepared using Illumina TruSeq and sequenced on Illumina sequence systems. Overall, the sequencing depth was between 26 and 159 million paired reads per sample.

To determine the abundance of functional microbial genetic groups, we used the KOunt pipeline [32], which aligns metagenomic sequences to the KEGG database. Within this pipeline, the complete metagenome sequencing data were trimmed for quality using Fastp [33] and assembled with MEGAHIT [34]. Proteins were predicted using Prodigal [35] and searched against the KEGG database (https://www.genome.jp/kegg/ko.html) (version 2020-10-04) [36] with KofamScan [37]. Then, BWA MEM [38] was used to map the reads to their assembly, and the abundance of KEGG orthologue, i.e. MG, was counted applying BamDeal [39] and BEDTools [40]. A more detailed description is provided in Mattock et al. [32] and Martínez-Álvaro et al. [30]. Initially, the abundances of 7976 MGs were identified. Only the 3632 MGs present in at least 70% of the samples with a mean relative abundance > 0.001% were retained for further analyses. These 3632 MGs accounted for 99.55% of the total counts identified in the dataset.

### Log-transformation

Zero values of the MG data were imputed to enable appropriate statistical analysis of these compositional data. Zero values in the microbiome dataset, most of which arise from sequencing detection limits or randomness in sequencing depth of samples rather than true absence, were replaced using the multiplicative replacement method (function cmultrepl) in the zCompositions package [41] in R. This imputation substitutes zeros with small positive values while preserving sample compositions and was needed to allow subsequent additive log-ratio (alr) transformation. Observed non-zero counts were proportionally rescaled to maintain the compositional nature of the original data. We applied alr transformation based on the following equation:

$${\mathrm{a}\mathrm{l}\mathrm{r}}_{\mathrm{M}\mathrm{G}\mathrm{i}}=\mathrm{l}\mathrm{n}\left(\frac{{\mathrm{x}}_{\mathrm{i}}}{{\mathrm{x}}_{\mathrm{r}\mathrm{e}\mathrm{f}}}\right)=\mathrm{ln}\left({\mathrm{x}}_{\mathrm{i}}\right)-\mathrm{ln}\left({\mathrm{x}}_{\mathrm{r}\mathrm{e}\mathrm{f}}\right),$$

where x_i_ is the relative abundance of the MG i and x_ref_ is the relative abundance of the reference MG. The reference MG used in the denominator of the alr transformation was selected following the procedure described in [30]. The MG ribulose-phosphate 3-epimerase [EC:5.1.3.1] (*rpe,* K01783) was selected as denominator because it best conserved the inter-sample Euclidean distances relative to centered log-ratio transformation, as evidenced by a high Procrustes correlation of 0.9974. In addition, the reference MG exhibited low log-ratio variance (0.0379), indicating that variability in each alr-transformed MG abundance was driven primarily and desirably by variation in the numerator MG rather than by the denominator [42].

### Variance component estimation

#### Estimation of genetic parameters of CH4P

A univariate Bayesian analysis was performed for CH4P to estimate its heritability, GEBVs, and selection response. The model fitted was as follows:

1 $$ {\mathbf{y}} = {\mathbf{Xb}} + {\mathbf{Zg}} + {\mathbf{e}}, $$

where **y** is a vector of observations of CH4P; **b** is a vector of fixed effects, including the combined effects of breed-diet-trial (17 classes), and age at entering the respiration chamber as covariable; vector **g** represents the random host genomic effects, and **X** and **Z** are incidence matrices relating vectors **b** and **g,** respectively, to the observations. The following (co)variance structures were assumed for **g** and the residual **e**:

2 $$\mathbf{g}|{\mathbf{G}}_{\mathrm{R}\mathrm{M}},{\upsigma}_{\mathrm{g}}^{2} \sim N(0,{\mathbf{G}}_{\mathrm{R}\mathrm{M}}{\upsigma}_{\mathrm{g}}^{2})$$

3 $$\mathbf{e}|\mathbf{I},{\upsigma}_{\mathrm{e}}^{2}\sim N(0,\mathbf{I}{\upsigma}_{\mathrm{e}}^{2})$$

where ${\upsigma}_{\mathrm{g}}^{2}$ and ${\upsigma}_{\mathrm{e}}^{2}$ are the host-genomic and residual variances, **I** is an identity matrix, and ${\mathbf{G}}_{\mathrm{R}\mathrm{M}}$ is the host-genomic relationship matrix among animals, which is calculated as

$${\mathbf{G}}_{\mathrm{R}\mathrm{M}}= \frac{{\mathbf{W}}^{^{\prime}}\mathbf{W}}{2{\sum}_{\mathrm{n}}^{1}{\mathrm{p}}_{\mathrm{n}}(1-{\mathrm{p}}_{\mathrm{n}})}$$

where the matrix **W** contains genotypes adjusted for twice the allele frequency, and where ${\mathrm{p}}_{\mathrm{n}}$ is the allele frequency for marker n in the population.

#### Estimation of genomic correlations among CH4P, production traits, and abundances of MGs

Host genomic, and phenotypic correlations among CH4P and production traits, namely DFI, ADG, and CCW, as well as abundances of MGs were estimated using Bayesian bivariate genomic analyses fitting the same fixed class effects and genomic effects as those in the univariate model Eq. (1). However, based on preliminary analyses, the age effect was replaced with a covariate of age at entering the experimental trial for DFI and age at slaughter for CCW as well as the abundances of MGs, whereas for ADG, the age covariate was nonsignificant and was therefore excluded from the model. Host genomic effects and residuals of the bivariate analyses were assumed to be distributed as follows:

4 $$\mathbf{g}|{\mathbf{G}}_{\mathrm{R}\mathrm{M}},{\mathbf{G}}_{0} \sim N(0,{\mathbf{G}}_{\mathrm{R}\mathrm{M}}\otimes{\mathbf{G}}_{0})$$

5 $$\mathbf{e}|\mathbf{I},{\mathbf{R}}_{0} \sim N(0,\mathbf{I}\otimes{\mathbf{R}}_{0})$$

where ${\mathbf{G}}_{0}$ and ${\mathbf{R}}_{0}$ are the 2 × 2 host genomic and residual (co)variance matrices between CH4P, production traits, and the abundances of MGs.

All variance component analyses were performed using Bayesian methodology implemented in the GIBBSF90 + program version 3.20 [43]. In multiple-trait analyses, GIBBSF90 + signs inverse-Wishart prior distributions to the (co)variance matrices of random effects by default, with low degrees of freedom, corresponding to weakly informative priors. In all analyses, convergence was tested using the POSTGIBBSF90 program version 3.15 by calculating the Z criterion of Geweke [44], which varied between −0.05 and 0.05 in univariate analyses and between −0.09 and 0.1 in bivariate analyses. Effective sample size of all parameters was greater than 180. Monte Carlo sampling errors were computed using time-series procedures and checked to be at least 10 times lower than the standard deviation of the marginal posterior distribution. Estimates of heritability, genetic, and phenotypic correlation were the means of their marginal posterior distributions for CH4P, production traits, and the abundances of MGs. As a credibility interval of the parameters, the highest posterior density interval at 95% probability (95HPD) was used. We also provide the posterior probability (Pr0) that a positive correlation estimated as posterior mean is > 0, or conversely that a negative correlation is < 0.

#### Identification of the most informative MGs for predicting CH4P

All bivariate analyses between the abundances of the 3631 alr-transformed MGs and CH4P were carried out to identify the most informative MGs. These were selected on the basis of the probability of the estimates of the genomic correlation being credible and the expected correlated response in CH4P when selecting for each MG, which was predicted as follows:

6 $${\mathrm{C}\mathrm{R}}_{\mathrm{C}\mathrm{H}4\mathrm{P}\mathrm{j}}=\frac{\mathrm{i}{*\mathrm{h}}_{\mathrm{j}}*{\mathrm{r}}_{\mathrm{g}\mathrm{C}\mathrm{H}4\mathrm{P}\mathrm{j}}*{\upsigma}_{\mathrm{g}\mathrm{C}\mathrm{H}4\mathrm{P}}}{{\overline{\mathrm{P}} }_{\mathrm{C}\mathrm{H}4\mathrm{P}}}*100,$$

where ${\mathrm{C}\mathrm{R}}_{\mathrm{C}\mathrm{H}4\mathrm{P}\mathrm{j}}$ is the correlated selection response in CH4P, expressed as a percentage of the phenotypic population mean (${\overline{\mathrm{P}} }_{\mathrm{C}\mathrm{H}4\mathrm{P}}$), after selection for the abundance of each MG j. For simplicity, the selection intensity i was chosen in all analyses to be equal to one, corresponding to a truncation selection proportion of approximately 38% of the population. In Eq. (6), ${\mathrm{h}}_{\mathrm{j}}$ is the square root of the MG’s estimated heritability, ${\mathrm{r}}_{\mathrm{g}\mathrm{C}\mathrm{H}4\mathrm{P}\mathrm{j}}$ is the estimated host genomic correlation between CH4P and the abundances of the MG j, and ${\upsigma}_{\mathrm{g}\mathrm{C}\mathrm{H}4\mathrm{P}}$ is the genetic standard deviation of CH4P. All genetic parameters were estimated as the mean of its marginal posterior distribution.

To identify MGs with sufficient impact for inclusion in the MDB strategy, we considered only those MGs in the multiple-trait model that had a ${\mathrm{r}}_{\mathrm{g}\mathrm{C}\mathrm{H}4\mathrm{P}}$ different from zero with Pr0 > 0.80 and resulted in a ${\mathrm{C}\mathrm{R}}_{\mathrm{C}\mathrm{H}4\mathrm{P}}$ exceeding 2% of its mean. Additional criteria were that the abundances of these MGs should have phenotypic and genomic correlations with CH4P in the same direction, with absolute phenotypic correlations greater than 0.1. Furthermore, MGs were required to have a relative abundance of at least 0.01%. A total of 140 MGs met all selection criteria. These candidate MGs were subsequently ranked by their ${\mathrm{C}\mathrm{R}}_{\mathrm{C}\mathrm{H}4\mathrm{P}}$ and those with their KEGG annotated biological function closely related to CH4P were chosen for MDB. Biological function considered as selection criteria included involvement in methanogenesis, hydrogen metabolism, redox balance, substrate provision for methanogens and general microbial growth potentially acting as hydrogen sink. We have chosen 43 of the 140 MGs based on these criteria, as preliminary analyses indicated that further MGs had little contribution to increase the accuracy of MDB. These will be referred to as MG43 throughout this manuscript. We conducted 903 (43 × 42 / 2) bivariate analyses to obtain the genomic and residual (co)variance matrix among the 43 MGs with highest informativeness to predict genomic CH4P. Genomic parameters were based on Markov chain Monte Carlo chains consisting of 1,000,000 iterations, with a burn-in period of 200,000, and to reduce autocorrelation every 100th sample was saved for inferences. The convergence was tested using the POSTGIBBSF90 as outlined earlier. From these analyses, we obtained the necessary genomic and residual co(variance) matrices used for the MDB strategy.

In a further MDB strategy, the aim was to reduce CH4P without compromising growth. Accordingly, we selected MGs that were negatively correlated with CH4P and positively correlated with ADG. Due to this restriction, selection criteria had to be relaxed to require the ${\mathrm{r}}_{\mathrm{g}\mathrm{C}\mathrm{H}4\mathrm{P}}$ and ${\mathrm{r}}_{\mathrm{g}\mathrm{A}\mathrm{D}\mathrm{G}}$ to be different from zero with Pr0 > 0.60 and the negative ${\mathrm{C}\mathrm{R}}_{\mathrm{C}\mathrm{H}4\mathrm{P}}$ and positive ${\mathrm{C}\mathrm{R}}_{\mathrm{A}\mathrm{D}\mathrm{G}}$ to exceed 1% of their respective means. Only further selection criterion was that the relative abundance of the MGs should be at least 0.01%. A total of 41 MGs met these criteria and no further selection based on biological function expected to be related to both CH4P reduction and growth improvement was applied. This decision was taken because the requirement for favourable effects on both CH4P and ADG indirectly resulted in the selection of MGs that are biologically highly relevant, such as those promoting increased microbial growth to act as a hydrogen sink and thereby reduce CH4 metabolism without compromising ADG. These MGs will be referred to as MG41. There were no overlapping MGs between the MG41 and MG43 sets. To obtain the host genomic and residual correlations among these 41 MGs, we conducted an additional 820 (41 × 40 / 2) bivariate analyses, using the same models and assumptions as described for MG43.

### Models for evaluation of genomic breeding values

Based on the results of the variance component analyses, the following evaluation models (E-Model) were used to compare selection scenarios aimed at reducing CH4P: using its direct measurement, MDB or combined with other performance traits. E-Model 1 was a univariate genomic evaluation of direct measurement of CH4P. E-Model 2–4 extended E-Model 1 by incorporating abundances of the most informative MGs (MG43 and MG41) to solely predict or to support estimation of GEBV_CH4P_. E-Model 5–9 additionally included key performance traits (DFI, ADG, CCW) to evaluate CH4P reduction and relevant correlated responses. Details of each model are given below:

E-Model 1: Uses directly measured CH4P as observations, including the same effects as described in Eq. (1) with (co)variance structures as outlined in Eqs. (2) and (3).

E-Model 2: Uses the abundances of the most informative MG43 as observations, with CH4P values set as missing to enable the estimation of its GEBVs based on genetic correlations with MG43 data. The model fitted the same effects as described in Eq. (1), with (co)variance structures defined in Eqs. (4) and (5). The ${\mathbf{G}}_{0}$ and ${\mathbf{R}}_{0}$ matrices had dimensions of 44 × 44, representing 43 MGs and CH4P. These matrices were based on the results of bivariate variance component analyses among these MGs and CH4P. For brevity, E-Model 2 is also referred to as MDB43.

E-Model 3: Uses both directly measured CH4P and the abundances of the most informative MG43 as observations, including the same effects as described in Eq. (1), with (co)variance structure as defined in Eqs. (4) and (5). The ${\mathbf{G}}_{0}$ and ${\mathbf{R}}_{0}$ matrices were of dimension 44 × 44 and identical to those used in E-Model 2.

E-Model 4: Uses the abundances of MG41, which are favourably associated with both CH4P and ADG, as observations, with CH4P and ADG values set as missing to enable the estimation of their GEBVs based on genetic correlations with MG41 observations. The model included the same effects as described in Eq. (1), with (co)variance structures as described in Eqs. (4) and (5). The ${\mathbf{G}}_{0}$ and ${\mathbf{R}}_{0}$ matrices had dimensions of 43 × 43, representing 41 MGs along with CH4P and ADG. These matrices were obtained based on results from bivariate variance component analyses between these MGs and both CH4P and ADG. For brevity, E-Model 4 is also referred to as MDB41.

E-Model 5: This model uses DFI, ADG, CCW, and CH4P as observations, including the same effects as described in Eq. (1), with (co)variance structures as outlined in Eqs. (4) and (5). The ${\mathbf{G}}_{0}$ and ${\mathbf{R}}_{0}$ matrices, both with dimensions 4 × 4, were developed based on estimates from bivariate variance component analyses among these traits. Additionally, a complementary sensitivity analysis was conducted based on E-Model 5, in which genetic correlations among production traits and with CH4P were replaced by estimates of phenotypic correlations. For production traits, the phenotypic correlations with CH4P were less than half of the corresponding estimated genetic correlations and were all below 0.4.

E-Model 6: This model uses the measures of the traits of DFI, ADG, CCW, and the abundances of MG43 as observations, with CH4P values set as missing to enable the estimation of its GEBVs based on genetic correlations with other traits and abundance observations of MGs, fitting the same effects as described in Eq. (1) with (co)variance structures as presented in Eqs. (4) and (5). The ${\mathbf{G}}_{0}$ and ${\mathbf{R}}_{0}$ matrices, each with dimensions 47 × 47, were obtained based on the results of bivariate variance component analyses among these traits and MG43.

E-Model 7: Uses the measures of all traits of DFI, ADG, CCW, CH4P, and the abundances of MG43 as observations, including the same effects as described in Eq. (1) with (co)variance structures as outlined in Eqs. (4) and (5). The ${\mathbf{G}}_{0}$ and ${\mathbf{R}}_{0}$ matrices, both with dimensions of 47 × 47, were identical to those used in E-Model 6.

E-Model 8: The model uses the measures of DFI, ADG, CCW and the abundances of MG41 as observations, with CH4P values set missing to obtain its GEBVs based on genetic correlations with other traits as well as MGs, fitting the same effects as described in Eq. (1) with (co)variance structures as outlined in Eqs. (4) and (5). The ${\mathbf{G}}_{0}$ and ${\mathbf{R}}_{0}$ matrices, both with dimensions of 45 × 45, were built based on the results of bivariate variance component analyses among these traits and MG41.

E-Model 9: This model uses the measures of all traits, DFI, ADG, CCW and CH4P as well as the abundances of MG41 as observations, including the same effects as described in Eq. (1) with (co)variance structures as presented in Eqs. (4) and (5). The ${\mathbf{G}}_{0}$ and ${\mathbf{R}}_{0}$ matrices, both with dimensions of 45 × 45, were identical to those used in E-Model 8.

The matrices were constructed using the mean values of the marginal posterior distributions of estimated (co)variance components from the corresponding bivariate analyses. To ensure consistency across all selection scenarios and models, the host genomic and residual variances of CH4P were kept the same, calculated as the average of 43 bivariate variance component analyses between CH4P and MG43. Based on the E-Models, GEBVs were obtained applying GBLUP analysis implemented in the GIBBSF90 + program version 3.20 [43], with (co)variance components fixed to the (co)variance components estimates obtained in this study. The GEBVs were obtained using Markov chain Monte Carlo sampling over 100,000 iterations, with a burn-in period of 20,000. To reduce autocorrelation between samples, the solutions of every 100th iteration were saved and used to estimate the GEBVs.

Finally, GEBV_RFI_ and GEBV_RCH4_ were derived from the GEBVs of DFI, ADG, CCW, and CH4P obtained with E-Model 5. GEBV_RFI_ and GEBV_RCH4_ were defined as the genetic residual of GEBV_DFI_ and GEBV_CH4P_ after accounting for their genetic relationships with GEBV_ADG_ and GEBV_CCW_, using genetic partial regression coefficients derived from their genetic (co)variance matrices [23, 45].

### Estimation of selection responses

We evaluated several selection scenarios aimed at reducing CH4P. The accuracy of the GEBVs of CH4P in each scenario was empirically estimated as the average of the individual accuracies obtained as follows:

$${\mathrm{A}\mathrm{c}\mathrm{c}\mathrm{u}\mathrm{r}\mathrm{a}\mathrm{c}\mathrm{y}}_{\mathrm{i}}= \sqrt{1-\frac{{\mathrm{s}\mathrm{d}}_{\mathrm{i}}^{2}}{{\mathrm{g}}_{\mathrm{R}\mathrm{M}\mathrm{i}\mathrm{i}}{\upsigma}_{\mathrm{g}\mathrm{C}\mathrm{H}4\mathrm{P}}^{2}}}$$

where ${\mathrm{s}\mathrm{d}}_{\mathrm{i}}$ is the standard deviation of the marginal posterior distribution of the host GEBVs for animal i and where ${\mathrm{g}}_{\mathrm{R}\mathrm{M}\mathrm{i}\mathrm{i}}$ is the diagonal element of ${\mathbf{G}}_{\mathrm{R}\mathrm{M}}$ for animal i.

Selection response was estimated as the difference between GEBV_CH4P_ mean of selected animals (using selection proportions of 5%, 10%, 20%, and 30%) and all animals in the population. For E-Models 1 to 3, GEBV_ADG_ were predicted in complementary analyses using a multiple-trait model that included ADG alongside CH4P, MDB43, or their combination, in order to obtain the correlated response in ADG when selection was based on any of the three CH4 mitigation strategies. For analyses based on E-Model 5, we also considered additional scenarios where selection of animals was based solely on GEBV_ADG_, GEBV_CCW_, GEBV_RFI_, or GEBV_RCH4_, using either the genetic parameters estimated in this study or assumed lower correlations to assess their impact.

## Results

Data were collected from beef cattle at the SRUC Beef and Sheep Research Centre. The animals showed an ADG of 1.476 kg (coefficient of variance (CV) = 0.19) with a DFI per day of 11.04 kg (CV = 0.12), an FCR of 7.61 kg feed/kg gain (CV = 0.17) and a mean CCW of 372.9 kg (CV = 0.10). Methane production averaged 190.5 ± 52.8 g/day, exhibiting the highest variability relative to its mean among all traits analysed (CV = 0.28, Additional file 1 Tables S1).

### Genomic and phenotypic parameters of production traits and CH4P

The estimated heritability for DFI was 0.61, which was the highest among all analysed production traits (Table 1). Interestingly, daily CH4P expressed a higher heritability (0.56) than ADG and CCW, though all posterior distributions had substantial overlap due to the size of the dataset. All these traits were positively correlated with each other at both the genetic and phenotypic levels, indicating unfavourable associations between production traits and CH4P. In particular, DFI showed strong positive genetic correlations with CH4P (0.88), ADG (0.93) and CCW (0.84). The growth-related traits ADG and CCW adjusted for age at slaughter exhibited undesirable moderate genetic correlations with CH4P, at 0.58 and 0.61, respectively. Phenotypic correlations were substantially lower than genetic correlations among production traits and with CH4P. While the estimated phenotypic correlation between CH4P and DFI approached a moderate level (0.39), those with ADG (0.22) and CCW (0.29) were low in magnitude.

**Table 1:** Estimated genetic parameters for beef production traits and methane production

Trait	Genetic variance	DFI	ADG	CCW	CH4P
DFI (kg/day)	0.790 (0.400–1.177)	0.61 (0.37–0.85)	0.93 (0.76–1.00)	0.84 (0.57–1.00)	0.88 (0.65–1.00)
ADG (kg/day)	0.020 (0.005–0.039)	0.56 (0.47–0.64)	0.39 (0.11–0.67)	0.73 (0.28–1.00)	0.58 (0.04–1.00)
CCW (kg)	294 (114–488)	0.60 (0.52–0.68)	0.28 (0.17–0.40)	0.48 (0.21–0.74)	0.61 (0.06–1.00)
CH4P (g/day)	838 (346–1489)	0.39 (0.27–0.51)	0.22 (0.10–0.35)	0.29 (0.16–0.42)	0.56 (0.26–0.86)

Posterior means of genetic variances, heritabilities (on diagonal), genetic correlations (above diagonal), and phenotypic correlations (below diagonal) including their 95% highest posterior density interval (in parentheses) for daily feed intake (DFI), average daily gain (ADG), cold carcass weight (CCW), and daily methane production (CH4P) using Bayesian analyses

### Comparison of expected selection responses of CH4P based on direct measurements and/or predicted by MDB

We evaluated the response to selection and accuracy of reducing CH4P in our population based on the GEBVs estimated using four different sources of information: CH4P itself (E-Model 1); the abundances of MG43, i.e. MDB43 (E-Model 2); the integration of CH4P and the abundances of MG43 (E-Model 3); and the abundances of MG41, i.e. MDB41 (E-Model 4), (Table 2).

**Table 2:** Responses in methane production using different microbiome-driven breeding strategies or directly measured methane

Selection intensity	Response	L.95HPD	H.95HPD	Pr0	Response (%)	L.95HPD (%)	H.95HPD (%)
*E-Model 1: Use of measured CH4P. Selection responses in CH4P (g/day), accuracy* = *0.63 (0.15)*							
2.06	−37.88	−50.91	−22.97	1.00	−19.89	−26.73	−12.06
1.76	−31.93	−40.20	−23.70	1.00	−16.76	−21.11	−12.44
1.40	−25.48	−31.71	−19.50	1.00	−13.38	−16.65	−10.24
1.16	−21.07	−26.27	−15.72	1.00	−11.06	−13.79	−8.25
*E-Model 2: Microbiome-driven breeding using the 43 most informative MGs genetically predicting CH4P. Selection responses in CH4P (g/day), accuracy* = *0.72 (0.03)*							
2.06	−44.28	−56.54	−32.42	1.00	−23.25	−29.68	−17.02
1.76	−33.56	−42.05	−24.94	1.00	−17.76	−22.13	−13.41
1.40	−24.51	−30.90	−19.10	1.00	−13.00	−16.41	−10.32
1.16	−19.63	−23.97	−15.32	1.00	−10.35	−12.60	−8.15
*E-Model 3: Integration of measured CH4P and microbiome-driven breeding using the 43 most informative MGs. Selection responses in CH4P (g/day), accuracy* = *0.89 (0.09)*							
2.06	−51.11	−57.51	−45.47	1.00	−26.83	−30.20	−23.87
1.76	−42.93	−48.18	−38.30	1.00	−22.54	−25.29	−20.11
1.40	−33.34	−37.24	−30.11	1.00	−17.50	−19.55	−15.81
1.16	−27.29	−30.07	−23.83	1.00	−14.33	−15.79	−12.51
*E-Model 4: Microbiome-driven breeding using the 41 most informative MGs favourably genetically correlated with both CH4P and ADG. Selection responses in CH4P (g/day), accuracy* = *0.61 (0.03)*							
2.06	−32.42	−45.05	−21.16	1.00	−17.02	−23.65	−11.11
1.76	−26.72	−37.35	−18.35	1.00	−14.03	−19.61	−9.64
1.40	−20.99	−26.83	−14.48	1.00	−11.02	−14.09	−7.60
1.16	−17.22	−21.55	−12.65	1.00	−9.04	−11.31	−6.64

Expected selection responses in methane production (CH4P, g/day) based on selection for reduced CH4P. Responses are based on either directly measured CH4P or microbiome-driven breeding using: (1) the abundances of the 43 most informative microbial genes (MGs) without any restrictions or (2) the abundances of the 41 most informative MGs favourably correlated with both CH4P and ADG

L.95HPD, lower limit of the 95% highest density interval; H.95HPD, upper limit of the 95% highest density interval; Pr0, probability of selection response being positive or negative; Response (%), selection response as a percentage of the phenotypic mean for CH4P (g/day)

Selection of animals with low GEBVs for CH4P estimated based on its direct measurements using respiration chambers resulted in an estimated response of −11.06% (95HPD: −13.79 to −8.25%) expressed as percentage of the phenotypic mean per generation at a selection intensity of 1.16. This estimated response increased to −19.89% (95HPD: −26.73 to −12.06%) at a higher selection intensity of 2.06. At this selection intensity, the higher accuracy obtained using MDB43 had an even greater estimated CH4P response of up to −23.25% (95HPD: −29.68 to −17.02%) per generation. The integration of directly measured CH4P with MDB43 data demonstrated the highest potential of all scenarios described in Table 2, indicating the largest estimated selection response in CH4P of −26.83% (95HPD: −30.20 to −23.87%) at i = 2.06. These findings highlight the value of functional MGs related to ruminal CH4P as a powerful source of information for genomic evaluations of CH4P. However, in the complementary analyses considering ADG, selection for reduced CH4 using directly measured CH4P, MDB43 or their integration led to significantly unfavourable correlated response in ADG ranging from −3.05% (95HPD: −4.83 to −1.06%) to −8.73% (95HPD: −12.48 to −4.04%) (Table 3).

**Table 3:** Correlated responses in growth using different microbiome-driven breeding strategies or directly measured methane aiming at reducing methane production

Selection intensity	Correlated response	L.95HPD	H.95HPD	Pr0	Correlated response (%)	L.95HPD (%)	H.95HPD (%)
*E-Model 1: Use of measured CH4P. Correlated responses in ADG (kg/day), accuracy* = *0.68 (0.03)*							
2.06	−0.11	−0.21	−0.01	0.99	−7.47	−14.00	−0.65
1.76	−0.09	−0.16	−0.03	1.00	−6.32	−10.65	−2.04
1.40	−0.07	−0.12	−0.03	1.00	−5.03	−8.02	−1.73
1.16	−0.06	−0.10	−0.02	1.00	−4.13	−6.77	−1.51
*E-Model 2: Microbiome-driven breeding using the 43 most informative MGs genetically predicting CH4P. Correlated responses in ADG (kg/day), accuracy* = *0.74 (0.02)*							
2.06	−0.11	−0.20	−0.03	1.00	−7.56	−13.26	−2.34
1.76	−0.10	−0.15	−0.05	1.00	−6.58	−10.47	−3.08
1.40	−0.07	−0.11	−0.03	1.00	−4.54	−7.24	−2.11
1.16	−0.05	−0.07	−0.02	1.00	−3.05	−4.83	−1.06
*E-Model 3: Integration of measured CH4P and microbiome-driven breeding using the 43 most informative MGs. Correlated responses in ADG (kg/day), accuracy* = *0.77 (0.03)*							
2.06	−0.13	−0.18	−0.06	1.00	−8.73	−12.48	−4.04
1.76	−0.12	−0.16	−0.07	1.00	−7.85	−10.58	−4.96
1.40	−0.10	−0.14	−0.07	1.00	−6.83	−9.20	−5.02
1.16	−0.09	−0.11	−0.06	1.00	−6.04	−7.46	−4.28
*E-Model 4: Microbiome-driven breeding using the 41 most informative MGs favourably genetically correlated with both CH4P and ADG. Correlated responses in ADG (kg/day), accuracy* = *0.58 (0.03)*							
2.06	0.037	−0.051	0.123	0.79	2.52	−3.46	8.32
1.76	0.021	−0.048	0.083	0.74	1.41	−3.24	5.60
1.40	0.000	−0.047	0.042	0.51	0.03	−3.18	2.83
1.16	0.003	−0.034	0.034	0.56	0.22	−2.30	2.20

Expected correlated responses in average daily gain (ADG, kg/day) based on selection for reduced CH4P. ADG genomic breeding values are predicted based on either directly measured CH4P or microbiome-driven breeding using: (1) the abundances of the 43 most informative microbial genes (MGs) without any restrictions or (2) the abundances of the 41 most informative MGs favourably correlated with both CH4P and ADG. Correlated response (%), correlated response as a percentage of the phenotypic mean for ADG (kg/day). For the explanations of other abbreviations used, see Table 2

Selection solely including MG41 information into MDB resulted in the lowest estimated response in CH4P ranging from −9.04% (at i = 1.16) to −17.02% (at i = 2.06) per generation (Table 2). In this scenario, we observed small, non-significant increases in ADG, as indicated by a probability being positive of less than 80% (Table 3). The accuracies of GEBVs, calculated as means within each scenario, were in line with the magnitude of the responses obtained across scenarios.

### Combining production traits with measured CH4P and/or MDB to improve selection for reduced CH4P

We evaluated the impact of integrating production traits with abundances of MGs and CH4P measurements on the accuracy of GEBVs and the expected selection responses for reduced CH4P (E-Models 5–9; Tables 4 and 5).

**Table 4:** Responses in methane production and growth using production traits and 43 most informative microbial genes

Selection intensity	Response	L.95HPD	H.95HPD	Pr0	Response (%)	L.95HPD (%)	H.95HPD (%)
*E-Model 5: Use of measured CH4P and production traits (DFI, ADG, CCW). Selection responses in CH4P (g/day), accuracy* = *0.79 (0.08)*							
2.06	−45.60	−58.87	−34.62	1.00	−23.94	−30.91	−18.18
1.76	−39.22	−47.00	−31.15	1.00	−20.59	−24.68	−16.35
1.40	−31.26	−35.76	−26.88	1.00	−16.41	−18.77	−14.11
1.16	−26.00	−29.22	−22.55	1.00	−13.65	−15.34	−11.84
*Correlated selection responses in ADG (kg/day), accuracy* = *0.73 (0.05)*							
2.06	−0.17	−0.25	−0.11	1.00	−11.68	−17.08	−7.34
1.76	−0.14	−0.19	−0.09	1.00	−9.67	−12.66	−6.08
1.40	−0.12	−0.15	−0.10	1.00	−8.42	−10.25	−6.59
1.16	−0.10	−0.12	−0.08	1.00	−6.96	−8.27	−5.50
*E-Model 6: Use of production traits with microbiome-driven breeding using the 43 most informative MGs to genetically predict CH4P. Selection responses in CH4P, accuracy* = *0.81 (0.06)*							
2.06	−53.98	−65.08	−41.66	1.00	−28.34	−34.17	−21.87
1.76	−44.03	−53.46	−32.35	1.00	−23.12	−28.07	−16.98
1.40	−33.37	−40.32	−25.72	1.00	−17.52	−21.17	−13.51
1.16	−26.87	−33.04	−19.62	1.00	−14.11	−17.35	−10.30
*Correlated selection responses in ADG (kg/day), accuracy* = *0.82 (0.09)*							
2.06	−0.25	−0.32	−0.18	1.00	−16.87	−21.88	−12.01
1.76	−0.21	−0.28	−0.15	1.00	−14.47	−18.91	−10.36
1.40	−0.16	−0.21	−0.12	1.00	−11.13	−14.52	−8.06
1.16	−0.12	−0.16	−0.07	1.00	−7.99	−10.81	−4.97
*E-Model 7: Use of production traits with integration of measured CH4P and microbiome-driven breeding using the 43 most informative MGs. Selection responses in CH4P, accuracy* = *0.90 (0.09)*							
2.06	−56.17	−63.51	−50.03	1.00	−29.49	−33.34	−26.27
1.76	−46.14	−51.94	−40.69	1.00	−24.22	−27.27	−21.37
1.40	−35.06	−40.15	−30.01	1.00	−18.41	−21.08	−15.75
1.16	−28.83	−33.40	−24.57	1.00	−15.14	−17.54	−12.90
*Correlated selection responses in ADG (kg/day), accuracy* = *0.83 (0.09)*							
2.06	−0.21	−0.27	−0.15	1.00	−14.34	−18.25	−10.36
1.76	−0.17	−0.22	−0.13	1.00	−11.67	−14.79	−8.48
1.40	−0.14	−0.18	−0.11	1.00	−9.31	−12.02	−7.13
1.16	−0.11	−0.14	−0.08	1.00	−7.46	−9.44	−5.62

Expected selection responses in methane production (CH4P, g/day) and correlated responses in average daily gain (ADG, kg/day) based on different selection scenarios including in the multiple-trait model: the production traits, directly measured CH4P and/or microbiome-driven breeding based on the 43 most informative microbial genes (MGs) associated with CH4P. Production traits additionally included in the multiple-trait model are daily feed intake (DFI), average daily gain (ADG) and age-adjusted cold carcass weight (CCW). For the explanations of other abbreviations used, see Table 2

**Table 5:** Responses in methane production and growth using production traits and the favourable 41 microbial genes

Selection intensity	Response	L.95HPD	H.95HPD	Pr0	Response (%)	L.95HPD (%)	H.95HPD (%)
*E-Model 8: Use of production traits (DFI, ADG, CCW) and microbiome-driven breeding using the 41 most informative MGs favourably correlated with both CH4P and ADG. Selection responses in CH4P (g/day), accuracy* = *0.77 (0.07)*							
2.06	−42.39	−51.39	−33.85	1.00	−22.25	−26.98	−17.77
1.76	−34.40	−41.91	−27.88	1.00	−18.06	−22.00	−14.64
1.40	−27.28	−32.44	−22.61	1.00	−14.32	−17.03	−11.87
1.16	−22.98	−26.55	−19.22	1.00	−12.06	−13.94	−10.09
*Correlated selection responses in ADG (kg/day), accuracy* = *0.78 (0.10)*							
2.06	−0.16	−0.21	−0.10	1.00	−10.64	−14.43	−6.65
1.76	−0.12	−0.16	−0.08	1.00	−7.99	−11.05	−5.08
1.40	−0.10	−0.13	−0.07	1.00	−6.93	−8.75	−5.06
1.16	−0.09	−0.11	−0.08	1.00	−6.36	−7.67	−5.17
*E-Model 9: As E-Model 8 with additional inclusion of measured CH4P. Selection responses in CH4P (g/day), accuracy* = *0.89 (0.09)*							
2.06	−51.62	−58.69	−43.86	1.00	−27.10	−30.81	−23.03
1.76	−43.70	−47.91	−39.40	1.00	−22.94	−25.15	−20.69
1.40	−34.35	−37.42	−31.66	1.00	−18.04	−19.64	−16.62
1.16	−28.48	−30.70	−25.85	1.00	−14.95	−16.12	−13.57
*Correlated selection responses in ADG (kg/day), accuracy* = *0.81 (0.10)*							
2.06	−0.17	−0.24	−0.11	1.00	−11.65	−15.98	−7.71
1.76	−0.16	−0.20	−0.12	1.00	−10.79	−13.37	−8.34
1.40	−0.10	−0.12	−0.07	1.00	−6.58	−8.10	−4.91
1.16	−0.08	−0.10	−0.06	1.00	−5.49	−6.89	−4.35

Expected selection responses in methane production (CH4P, g/day) and correlated responses in average daily gain (ADG, kg/day) based on different selection scenarios including in the multiple-trait model the production traits, directly measured CH4P and/or microbiome-driven breeding based on the 41 most informative microbial genes (MGs) favourably genetically correlated with both CH4P and ADG. Production traits additionally included in the multiple-trait model are daily feed intake (DFI), average daily gain (ADG) and age-adjusted cold carcass weight (CCW). For the explanations of other abbreviations used, see Table 2

Using DFI, ADG, and CCW together with CH4P data improved the accuracy of GEBV_CH4P_ in comparison to using CH4P measurements alone, from 0.63 ± 0.15 (Table 2) to 0.79 ± 0.08 (Table 4). DFI, ADG, and CCW showed similar accuracies of 0.83 ± 0.06, 0.73 ± 0.05, and 0.75 ± 0.05, respectively. When CH4P data were set as missing, the integration of production traits with MG43 abundances achieved an accuracy of 0.81 ± 0.06, slightly higher than 0.77 ± 0.07 obtained using MG41, which included only MGs favourably genetically correlated with both ADG and CH4P (Tables 4 and 5). Again, these accuracies were substantially higher than those obtained without considering production traits. The integration of production traits and informative MGs gave a higher expected selection response compared to combining them with measured CH4P data. At i = 2.06, response to selection of −28.34% (95HPD: −34.17 to −21.87%) per generation were obtained with the integration of production traits and MG43 abundances, whereas the integration with measured CH4P data led to a response of −23.94% (95HPD: −30.91 to −18.18%). Notably, the combination of production traits, CH4P, and MG43 abundances demonstrated the highest accuracy among those in Table 4, at 0.90 ± 0.09 and an expected CH4P response of −29.49% (95HPD: −33.34 to −26.27%) per generation at i = 2.06.

When production traits were integrated with MDB41, using the 41 MGs favourably correlated with CH4P and ADG, the expected selection responses were lower than those of the strategies without this restriction (MDB43; Table 5 vs. 4). An even greater drawback of the integration of production traits and MDB41 was the highly relevant (Pr0 = 1) reduction in ADG, indicating substantial genetic correlations between production traits and CH4P, which counteracts the desired effect of MG41 in improving ADG.

### Selection responses in production and methane traits including sensitivity analyses using assumed low genetic correlations

**Fig. 1:**
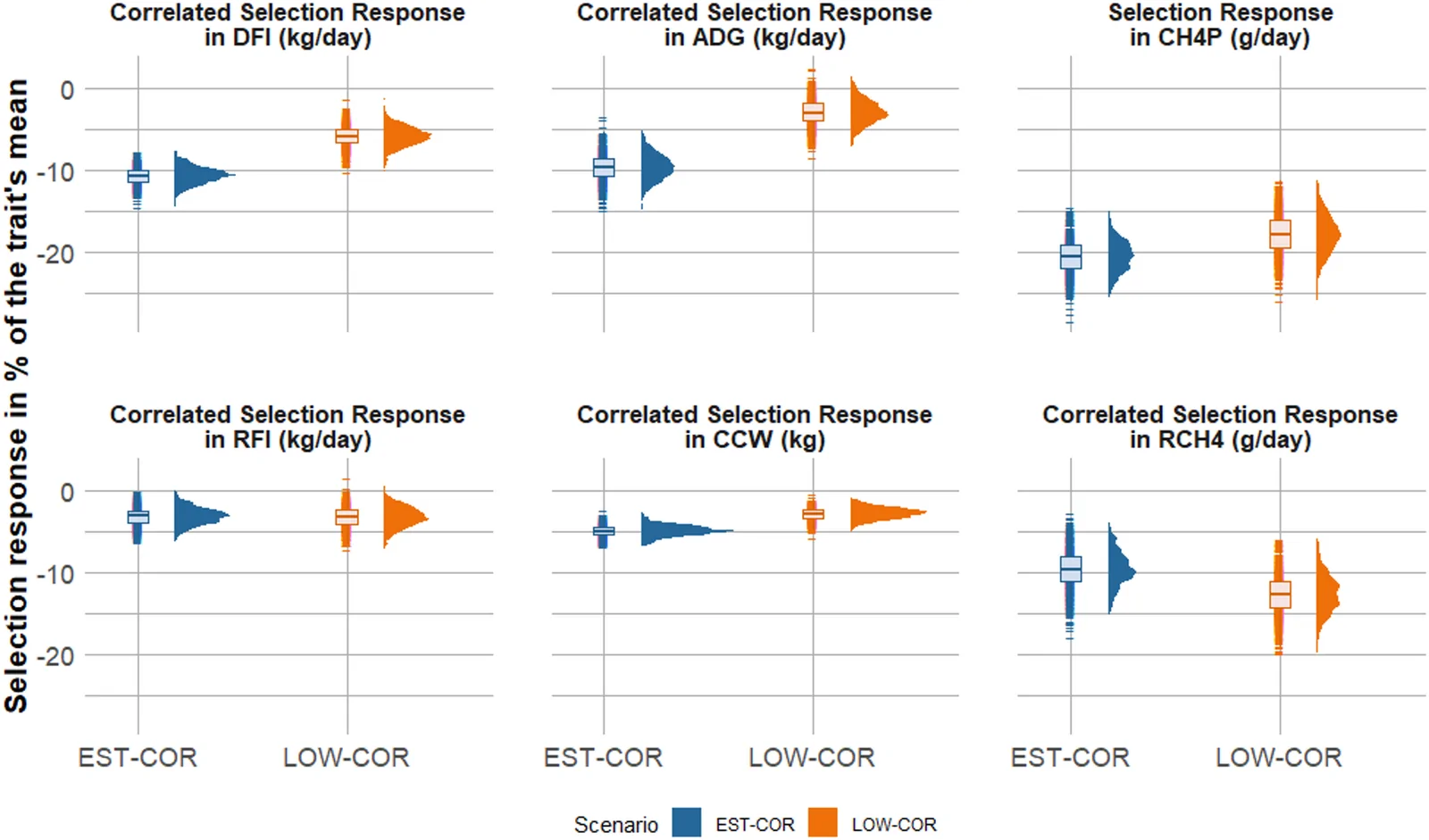
Selection scenario for reduced methane production. Direct and correlated selection responses under the selection scenario for reduced daily methane production (CH4P), based on either the high estimated genetic correlations between traits (EST-COR, blue) or the assumed low genetic correlations equivalent to phenotypic correlations (LOW-COR, orange). Results included direct response in CH4P, as well as correlated responses to selection in daily feed intake (DFI), average daily gain (ADG), cold carcass weight (CCW), residual feed intake (RFI), and residual methane production (RCH4). A selection intensity of 1.755 was applied, corresponding to selecting the best 10% of the population. Means, first quantiles and the whole estimated marginal posterior distributions of the selection responses for each trait and correlation scenario are presented

**Fig. 2:**
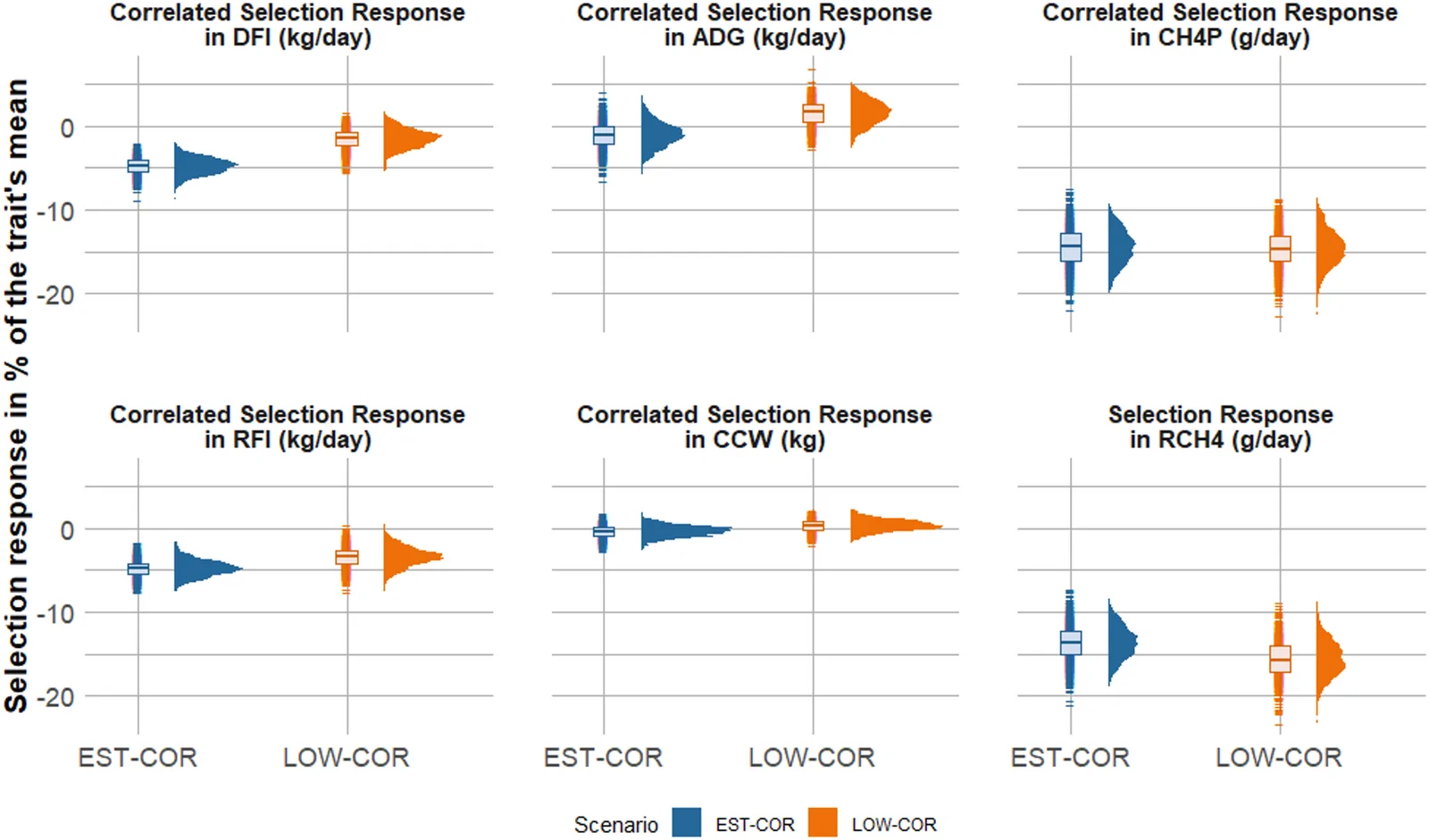
Selection scenario for reduced residual methane production. Direct and correlated selection responses under the selection scenario for reduced residual daily methane production (RCH4), based on either the high estimated genetic correlations between traits (EST-COR, blue) or the assumed low genetic correlations equivalent to phenotypic correlations (LOW-COR, orange). Results included direct response in RCH4, as well as correlated responses to selection in daily feed intake (DFI), average daily gain (ADG), cold carcass weight (CCW), residual feed intake (RFI), and methane production (CH4P). A selection intensity of 1.755 was applied, corresponding to selecting the best 10% of the population. Means, first quantiles and the whole estimated marginal posterior distributions of the selection responses for each trait and correlation scenario are presented

**Fig. 3:**
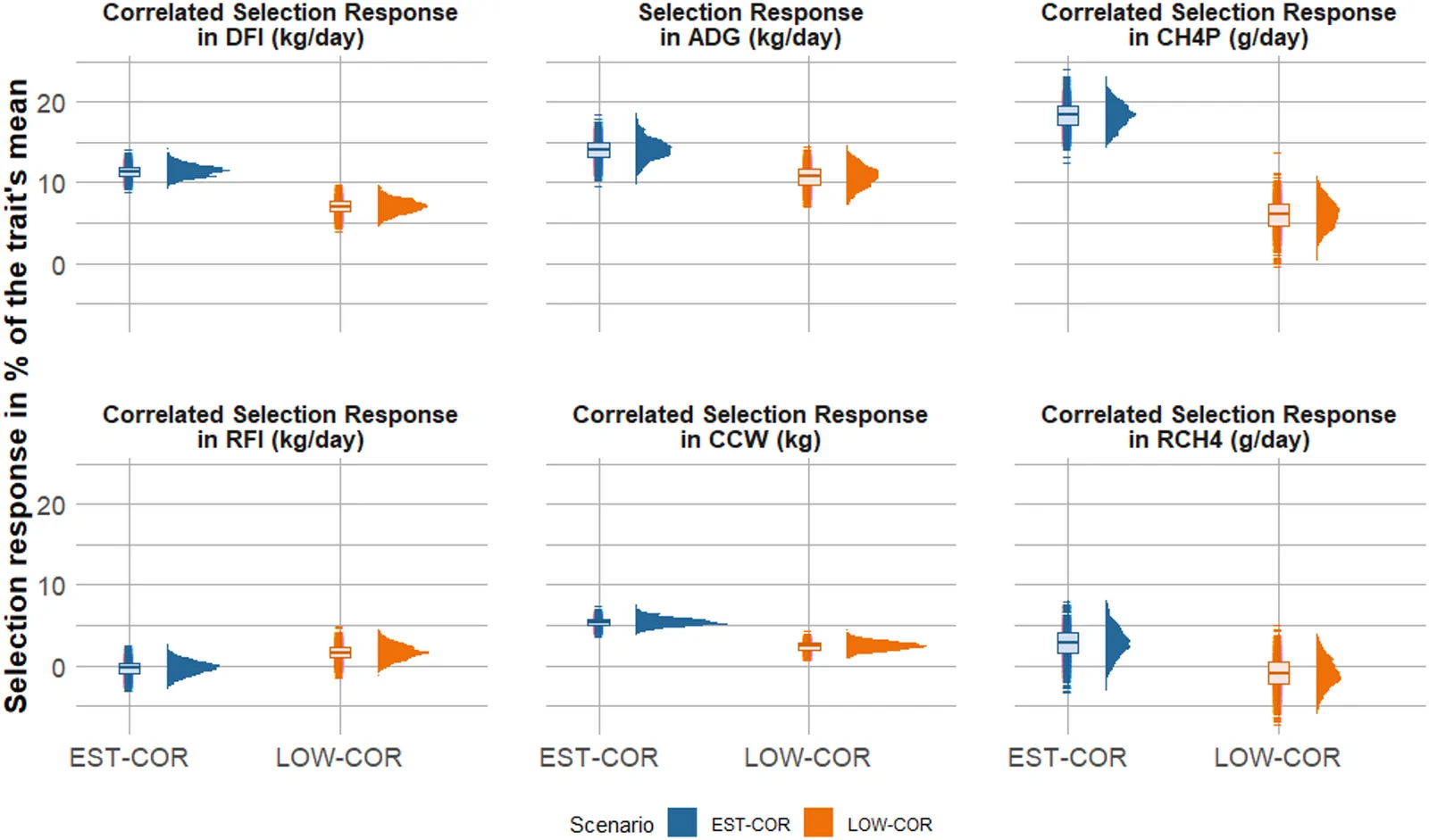
Selection scenario for improved average daily gain. Direct and correlated selection responses under the selection scenario for improved average daily gain (ADG), based on either the high estimated genetic correlations between traits (EST-COR, blue) or the assumed low genetic correlations equivalent to phenotypic correlations (LOW-COR, orange). Results included direct response in ADG, as well as correlated responses to selection in daily feed intake (DFI), cold carcass weight (CCW), residual feed intake (RFI), methane production (CH4P), and residual methane production (RCH4). A selection intensity of 1.755 was applied, corresponding to selecting the best 10% of the population. Means, first quantiles and the whole estimated marginal posterior distributions of the selection responses for each trait and correlation scenario are presented

**Fig. 4:**
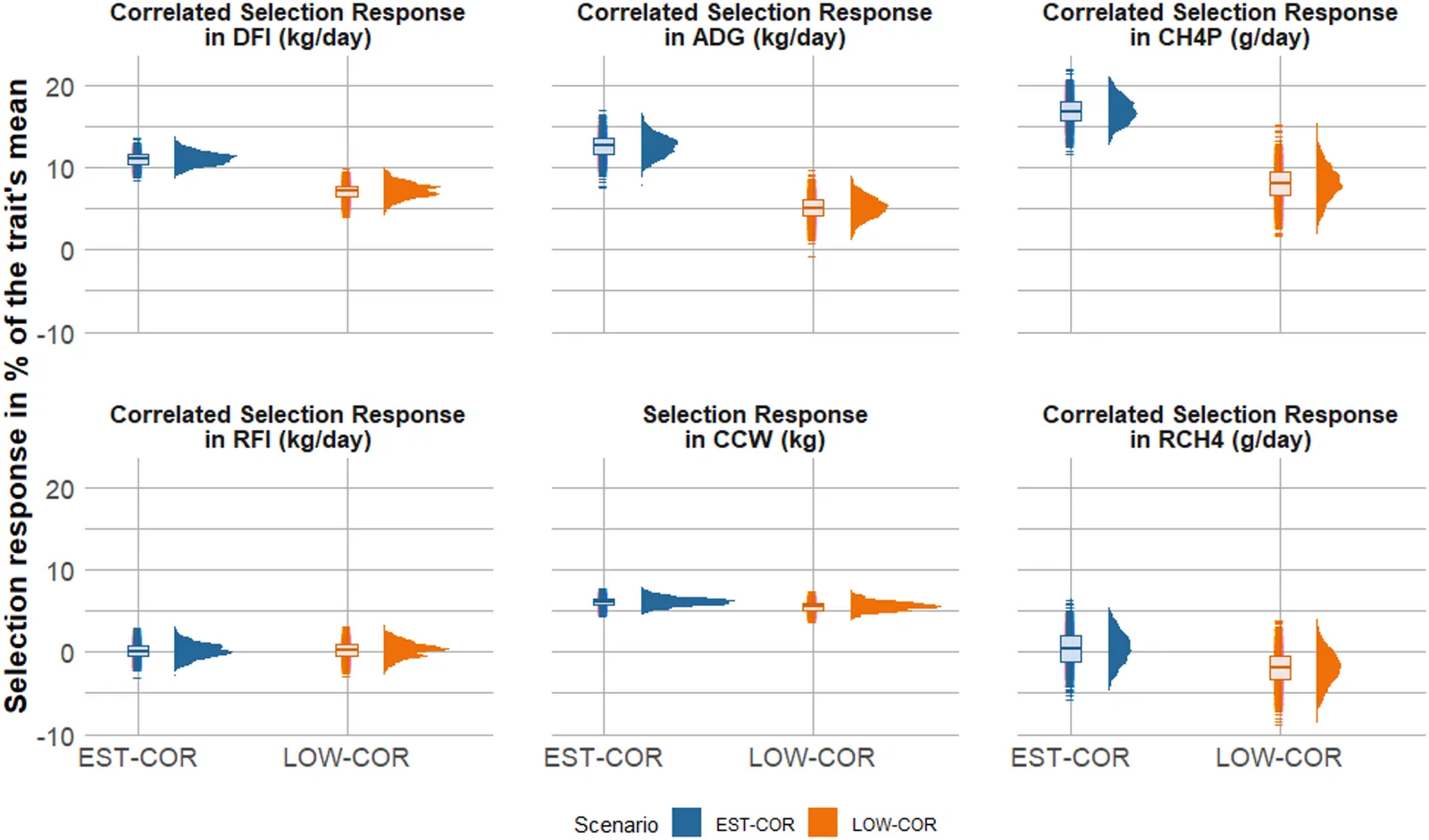
Selection scenario for improved age-adjusted cold carcass weight. Direct and correlated selection responses under the selection scenario for improved age-adjusted cold carcass weight (CCW), based on either the high estimated genetic correlations between traits (EST-COR, blue) or the assumed low genetic correlations equivalent to phenotypic correlations (LOW-COR, orange). Results included direct response in CCW, as well as correlated responses to selection in daily feed intake (DFI), average daily gain (ADG), residual feed intake (RFI), methane production (CH4P), and residual methane production (RCH4). A selection intensity of 1.755 was applied, corresponding to selecting the best 10% of the population. Means, first quantiles and the whole estimated marginal posterior distributions of the selection responses for each trait and correlation scenario are presented

**Fig. 5:**
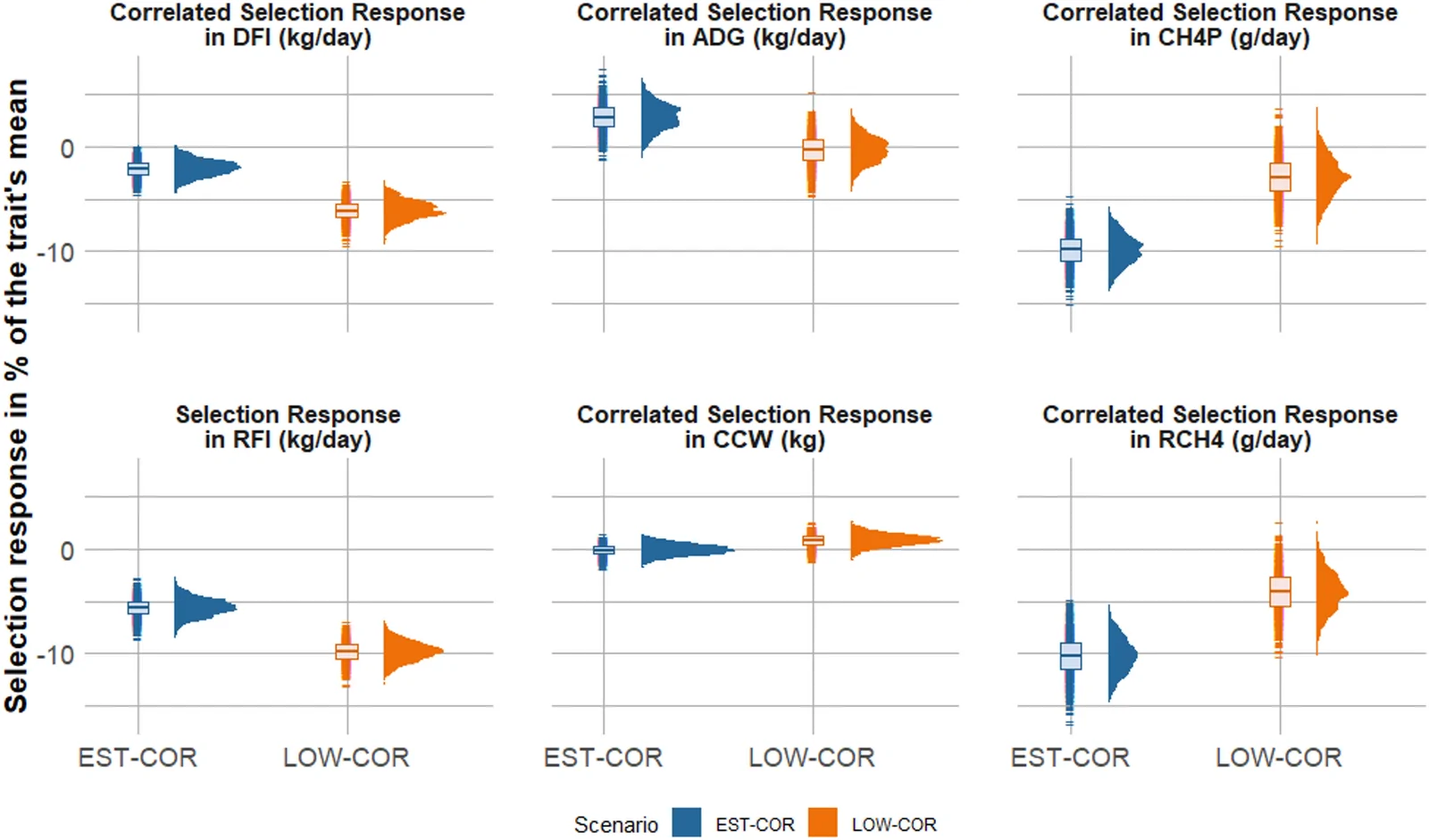
Selection scenario for reduced residual feed intake. Direct and correlated selection responses under the selection scenario for reduced residual feed intake (RFI), based on either the high estimated genetic correlations between traits (EST-COR, blue) or the assumed low genetic correlations equivalent to phenotypic correlations (LOW-COR, orange). Results included direct response in RFI, as well as correlated responses to selection in daily feed intake (DFI), average daily gain (ADG), cold carcass weight (CCW), methane production (CH4P), and residual methane production (RCH4). A selection intensity of 1.755 was applied, corresponding to selecting the best 10% of the population. Means, first quantiles and the whole estimated marginal posterior distributions of the selection responses for each trait and correlation scenario are presented

Figures 1, 2, 3, 4 and 5 illustrate the expected selection responses using different selection strategies based on various traits: CH4P, RCH4, ADG, CCW and RFI. Each strategy targets selection of the top 10% of the population and responses are evaluated under two scenarios: (1) based on the high genetic correlations among traits, as estimated from our data, and (2) based on the assumed low genetic correlations equivalent to the lower phenotypic correlations estimated from our data. For this analysis we introduced two new traits, RCH4 and RFI, which are specifically derived to be genetically uncorrelated with ADG and CCW. This approach ensures that selection on these new traits does not inadvertently affect production traits.

Direct selection for CH4P resulted in a substantial expected selection response of −20.59% (95HPD: −24.68 to −16.35%) per generation (Fig. 1, Table S2), highlighting the high potential to reduce CH4P through breeding. However, this selection strategy also led to substantially unfavourable correlated responses in ADG of −9.67% (95HPD: −12.66 to −6.08%) and CCW of −4.93% (95HPD: −6.51 to −3.72%). These declines in ADG and CCW were also associated with a substantial adverse response in DFI of −10.70% (95HPD: −12.65 to −8.58%) when using the high estimated genetic correlations obtained in our population. The decrease in DFI resulted in an improvement in feed efficiency as indicated by a negative RFI value of −3.14% (95HPD: −5.22 to −1.05%). Even when assuming a low genetic correlation between ADG and CH4P (r_gADG,CH4P_ = 0.22), there was still a meaningful adverse response in ADG of −2.95% (95HPD: −5.89 to 0.48%, Pr0 = 0.96) (Table S7). Correlated response in RCH4 of −9.65% (95HPD: −14.46 to −4.93%; Table S2) was about half of that based on directly measured CH4P when using high estimated genetic correlations but increased to −12.65% (95HPD: −17.29 to −7.80%; Table S7) when using the assumed low genetic correlations.

Given the high genetic correlations among traits, the selection on RCH4 led to selection responses that were approximately 35% lower than those from breeding for directly measured CH4P (−13.68% (95HPD: −17.67 to −9.29%)) (Fig. 2, Table S3). As expected, this strategy showed small correlated responses on ADG and CCW with their 95HPD including zero, and the reduction in DFI was about a half (estimates of −4.81% (95HPD: −6.71 to −2.88%)) of that obtained by selection for directly measured CH4P. Using the assumed low genetic correlations among traits, selection for RCH4 substantially improved the response in CH4 mitigation but was still lower than that obtained for direct selection on measured CH4P. This finding indicates that even low positive genetic correlations between production traits and CH4P substantially restrict the potential to reduce CH4P when using a selection trait that prohibit adverse effects on growth traits.

Selection for ADG gave rise to a substantial estimated improvement of 14.11% (95HPD: 11.39–16.80%) (Fig. 3, Table S4) in percentage of the trait mean. This selection also resulted in an increase in estimated DFI of 11.44% (95HPD: 9.92–13.03%) and was associated with a strong unfavourable increase in CH4P of 18.30% (95HPD: 15.05–21.30%).

**Fig. 6:**
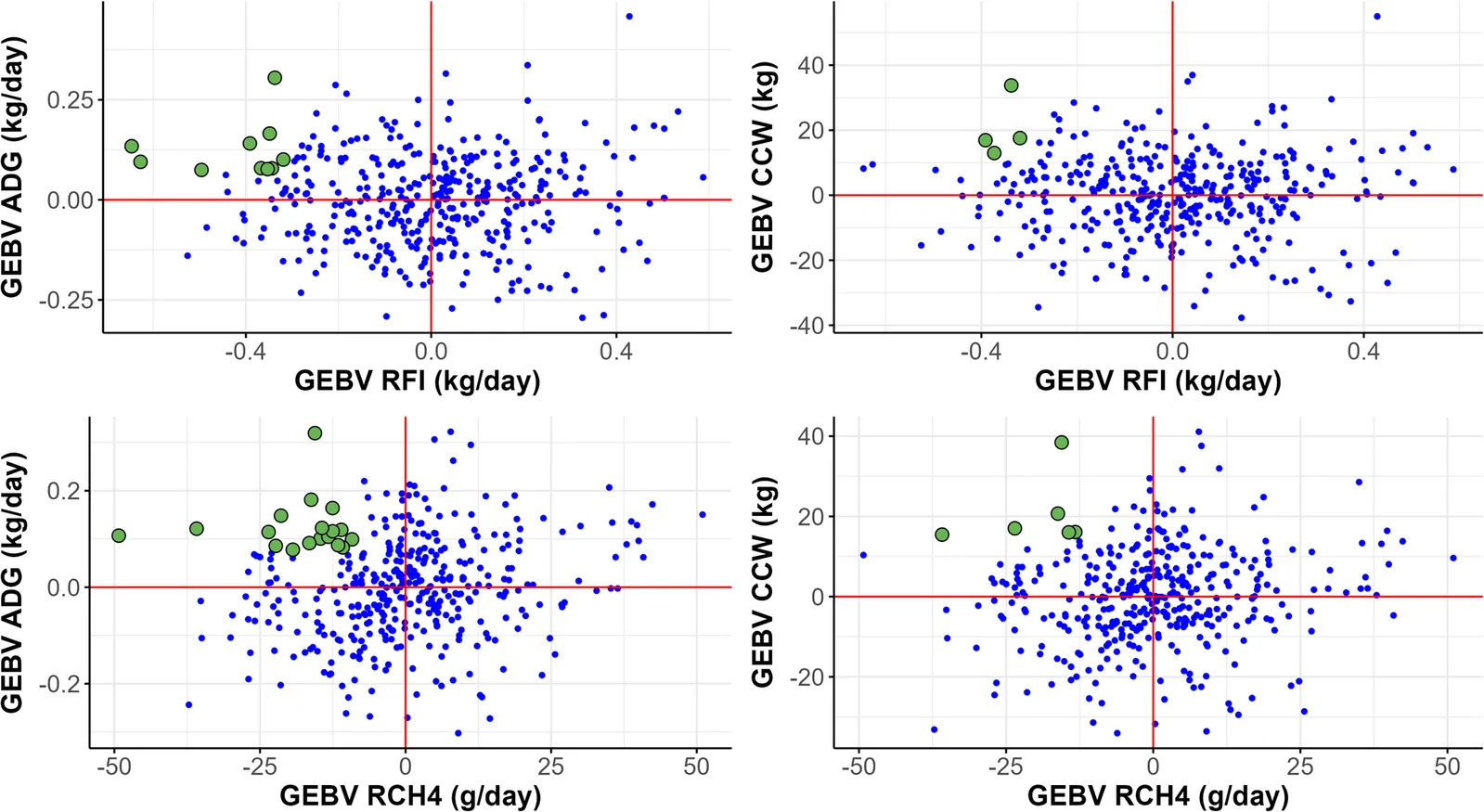
Selection of animals with correlation-breaking favourable GEBVs. The scatter plot illustrates the relationship between genomic estimated breeding values (GEBVs) for residual feed intake (RFI) or residual methane production (RCH4) (x-axis) and average daily gain (ADG) or cold carcass weight (CCW) (y-axis). Green-highlighted dots indicate animals with favourable GEBVs for both traits, i.e., positive GEBVs for ADG or CCW combined with negative GEBVs for RFI or RCH4. Highlighted animals achieved simultaneously a minimum improvement of 5% (relatively to the phenotypic means) in these traits

Selection for RFI using the high estimated genetic correlations showed similar directions of expected direct and correlated responses across most traits, as did selection for RCH4 (Fig. 5, Table S6). In particular, selection for reduced RFI resulted in a substantial estimated reduction in RCH4_,_ reflecting the high estimated genetic correlations between both traits and DFI. Assuming low genetic correlations of DFI with growth traits resulted in a significant rise in estimated selection response for RFI of −9.80% (95HPD: −11.75 to −7.95%), compared with the scenario using high correlations, which yielded −5.63% (95HPD: −7.33 to −3.84%) (Table S11 vs. S6). To illustrate the opportunity to select animals, with low GEBVs of RFI or RCH4 and high GEBVs for ADG and CCW, these residual traits were plotted against ADG and CCW in Fig. 6, considering the strong estimated genetic correlations among production traits and with CH4P. GEBVs of animals are highlighted that achieved in both traits a minimum improvement of 5% relatively to their corresponding phenotypic means. Summaries of the selection responses for all the selection scenarios with different selection intensities are shown in Additional file 1 Tables S2-11.

## Discussion

Animal breeding has been identified as a promising strategy for mitigating CH4P from ruminant livestock. However, relatively few studies have reported genetic correlations between CH4P and economically important production traits or developed cost-effective selection strategies suitable for practical implementation. These gaps contribute to uncertainty around the feasibility of practical breeding for reduced CH4P, particularly concerning potential adverse effects on production traits and high costs associated with direct measurement. In the present study, we aimed to address these challenges by utilising CH4P measurements obtained through the gold standard measurement technique, respiration chambers, to estimate the genetic parameters of CH4P and three key production traits, DFI, ADG, and CCW. We also derived selection criteria for reducing CH4P that do not adversely affect production traits based on their genomic evaluation in a multiple-trait model and, alternatively, based on the MDB strategy.

The heritabilities of production traits in finishing steers in our study were 0.61 (95HPD: 0.37–0.85) for DFI, 0.39 (95HPD: 0.11–0.67) for ADG, and 0.48 (95HPD: 0.21–0.74) for CCW, which were similar to those recently reported for finishing beef cattle. For example, heritabilities from other studies ranged from 0.28 ± 0.09 to 0.54 ± 0.13 for DFI and from 0.11 ± 0.06 to 0.54 ± 0.13 for ADG [46–48], with the exception of [49], who estimated a higher DFI heritability of 0.84 ± 0.12. The estimated heritability for CCW in our study also aligns with values reported across various studies and breeds, which range from 0.28 ± 0.02 to 0.51 ± 0.03 [50–54]. Our estimated genetic correlation between DFI and ADG was high at 0.93 (95HPD: 0.76 to 1.00), placing it at the upper end of the range reported in the literature (0.34 ± 0.10 to 0.86 ± 0.07) [47, 49]. Genetic correlations between DFI and CCW, and between ADG and CCW, were consistent with the literature, ranging from 0.65 ± 0.11 to 0.73 ± 0.07 and from 0.29 ± 0.19 to 0.94 ± 0.01, respectively [46, 54–56].

The heritability of CH4P (g CH4/day, respiration chambers) estimated in this study was 0.56 (95HPD: 0.26–0.86), which is higher than previously reported values for beef cattle. These values were based on respiration chamber measurements between 0.21 ± 0.11 and 0.30 ± 0.06 [19, 20, 57, 58]), and using the GreenFeed system at 0.43 ± 0.11 [59]. In dairy cattle, mostly CH4 measurements based on sniffer techniques were used, and heritability estimates ranged from 0.12 ± 0.04 to 0.45 ± 0.11 [60–62], whereas those based on GreenFeed measures were consistently moderate at 0.31 ± 0.15 and 0.36 ± 0.12 [63, 64]. In sheep, Pinares-Patiño et al. [65] estimated a heritability of 0.29 ± 0.05 using respiration chambers. Importantly, all these heritability estimates are population-specific and influenced by various environmental, technological and methodological factors. In our research, the high heritability estimate for CH4P is most likely due to the controlled environmental conditions, known dietary composition, standardised husbandry and uniform use of respiration chambers at the SRUC’s Beef research farm, where the animals were born and raised. In contrast, estimates of heritability are mostly likely lower in studies involving beef cattle that grazed on pasture throughout their lives except for the period of CH4P measurement, including transportation, acclimatisation, and measurements of CH4P in respiration chambers at testing facility [19, 20, 57]. Moreover, differences can be explained in part by recording methods as well as differences in recording time length [59, 66, 67]. Ryan et al. [59] found that the heritability estimates for CH4 emissions increased with the length of recording time when GreenFeed was used, suggesting that extended measurement periods reduce random environmental variability and thus the denominator of the heritability.

The genetic correlation between CH4P and DFI has been reported to be 0.84 ± 0.06 and 0.83 ± 0.05 in beef cattle [19, 20] and ranges from 0.42 ± 0.30 to 0.83 ± 0.11 in dairy cattle [21, 64, 68]. The estimates reported for beef cattle align with those in the current study, confirming a strong genetic association between CH4P and DFI across different populations. Furthermore, Donoghue et al. [19] estimated a correlation of 0.79 ± 0.08 between finishing weight and CH4P, which is slightly higher than the correlation of 0.61 (95HPD: 0.06–1.00) estimated between CCW and CH4P in this study. Phenotypic correlations between ADG and CH4P have been reported to range from 0.15 ± 0.01 to 0.44 ± 0.26 [69–73], which aligns with our estimated phenotypic correlation of 0.22 (95HPD: 0.10–0.35). However, only a limited number of studies have estimated genetic correlations between ADG and CH4P, indicating a significant gap in the literature. Addressing this gap is essential for developing breeding strategies that effectively mitigate CH4P while maintaining or improving growth traits. Uemoto et al. [74] reported a genetic correlation estimate of 0.82 ± 0.03 between ADG and predicted daily CH4P, based on DFI, body weight, total digestible nutrients, and roughage ratio in Japanese Black steers, which is higher than our estimate of 0.58 (95HPD: 0.04–1.00).

Utilising the estimated genetic parameters, we subsequently evaluated the expected selection responses through various breeding strategies targeting different traits. Furthermore, in sensitivity analyses, we used the phenotypic correlations as proxies for the genetic correlations because they were substantially lower than the estimated genetic correlations and thus provided the required range of parameter values. Consequently, the results offer clear insight into the impact of the genetic correlations on the selection responses of production traits when selection is applied to CH4 emissions or vice versa. Selection of the best 10% of the population based on GEBVs of directly measured CH4P obtained in a univariate analysis indicated an estimated reduction of 17% per generation. This suggests that CH4P can be efficiently improved through selection, due to its large genetic variation, most likely because it was never under intense natural or artificial selection, either directly or indirectly. Even further reductions in measured CH4P were achieved by using it in a multiple-trait genetic evaluation model with production traits. Due to their strong genetic correlations, this approach increased the accuracy of GEBV_CH4P_ from 0.63 ± 0.15. to 0.79 ± 0.08. This selection for CH4 mitigation revealed that a decrease in DFI was the main driver for reduced CH4P, with the consequences of a high negative response in ADG of −9.67% (95HPD: −12.66 to −6.08%) and age-adjusted CCW of −4.93% (95HPD: −6.51 to −3.72%) per generation. Therefore, GEBV_RCH4_ were derived from the GEBVs of CH4P genetically adjusted for ADG and CCW to obtain a selection criterion which reduces CH4P without compromising the production traits. GEBV_RCH4_ was estimated using genetic partial regression coefficients derived from the genetic (co)variance matrix of CH4P, ADG and CCW [23, 45]. This approach can be adapted to any other population with different traits in their breeding objective being unfavourably genetically correlated to CH4P (e.g. in dairy cattle). The RCH4 derived at genetic level is likely more advantageous than that obtained at the phenotypic level, which may not be genetically independent of production traits [22, 75]. Another benefit of selection on RCH4 was that the correlated reduction in DFI did not lead to a decline in production traits. This finding indicates that the use of RCH4 to reduce CH4P served indirectly as a strategy to optimise feed intake at a level that did not negatively affect production traits.

Similarly, GEBV_RFI_ was derived from GEBV_DFI_ and GEBVs for ADG and CCW. Since DFI and CH4P were highly genetically correlated in our population, RFI is a good candidate for cost-effective practical breeding, enabling improvement of feed efficiency with substantial change in CH4P of −9.91% (95HPD: −13.20 to −7.01%), without the expense of measuring CH4 emissions. In contrast, the results, which are based on the assumed low genetic correlations between DFI and production traits or CH4P, indicate that selection for RFI or RCH4 differed in their responses; thus, selection for both traits would be necessary to achieve meaningful improvements in both feed efficiency and CH4P reduction. Another finding was that the selection responses for RFI and RCH4_,_ based on the high genetic correlations estimated in our population, were lower than those obtained in the sensitivity analysis using assumed low genetic correlations. These lower responses, in the case of the strong correlations between DFI with CH4P and the production traits, can also be explained by the small variation left in the GEBV_RFI_ and GEBV_RCH4_ after adjustment for the GEBVs of ADG and CCW. In contrast, the sensitivity analysis assumed low genetic correlations, which resulted in greater variations in GEBV_RFI_ and GEBV_RCH4_, leading to higher selection responses for these traits. In other selection scenarios, targeting either reduced CH4P or improved ADG or CCW, the use of the assumed low genetic correlations between CH4P and production traits declined their selection responses. These declines were due to lower accuracies resulting from lower correlations among these traits.

By plotting the GEBVs of RFI or RCH4 against those of ADG or CCW, we identified animals in our data with desirable low RFI or RCH4 values that also had meaningful high GEBVs for ADG or CCW (Fig. 6), despite the absence of correlation between these residual traits and production traits. Selection of such cattle is recommended for sustainable breeding.

A further challenge for cost-effective selection on CH4P is the need to measure it continuously on a large cohort of animals to achieve accurate GEBVs. Recently, Martínez-Álvaro et al. [13] developed a cost-effective methodology to predict CH4Y based on the composition of the rumen microbiome. In contrast, in this study, we identified the 43 MGs that were most informative to predict CH4P, based on their expected correlated selection responses. These MGs were entirely different from those identified in the previous study for CH4Y [13], indicating that CH4Y and CH4P represent distinct traits regarding the key MGs involved in ruminal biological processes. The results indicated that MDB based on 43 MGs gave similar expected selection responses compared to using direct CH4P measurements, with a slightly higher accuracy of 0.72 ± 0.03 vs. 0.63 ± 0.15 and selection responses of −17.76% (95HPD: −22.13 to −13.41%) vs. −16.76% (95HPD: −21.11 to −12.44%). The slight increase in accuracy using MG43 could be due to the close biological functions of the MGs with CH4 metabolism [76].

The advantage of MDB is that the functions of the selected 43 MGs are well characterised. The majority of MGs positively correlated with CH4P are involved in oxidoreductase processes, catalysing redox reactions and contribution to the biosynthesis of essential cofactors such as nicotinamide adenine dinucleotide (NAD) and ferredoxin. For example, *rnfD* regulates the enzyme H⁺/Na⁺-translocating ferredoxin:NAD⁺ oxidoreductase subunit D, which catalyses the electron transfer between NAD and ferredoxin [77]. In their reduced forms, cofactors such as reduced ferredoxin and NADH can donate electrons to H⁺, leading to the production of H_2_ via hydrogenases, or they can serve as electron donors in various metabolic pathways that contribute to methanogenesis [6, 78]. The conversion of NAD⁺ to its reduced form (NADH) is facilitated by the enzyme L-lactate dehydrogenase encoded by *lldE* [79]. Similarly, reduced ferredoxin is generated by the oxidative decarboxylation of 2-oxoglutarate to succinyl-CoA, a reaction catalysed by the enzyme 2-oxoglutarate ferredoxin oxidoreductase, encoded by *oorD* [80]. Ferredoxin oxidoreductase has been reported to be more abundant in high CH4-emitting beef cattle [81]. In relation to redox balance, the KEGG orthologue K03811, corresponding to the nicotinamide mononucleotide (NMN) transporter, exhibited the strongest correlation with CH4P. This transporter facilitates the uptake of NMN from the environment into cells, where NMN serves as a key intermediate in the biosynthesis of NAD [82, 83]. Additionally, the MG *sufE* is involved in the biosynthesis of iron- sulphur clusters, which include electron carriers such as ferredoxins [84]. Moreover, *PANK1_2_3* are involved in CoA synthesis [85], while *menI* catalyses the synthesis of vitamin K, which is coupled with the release of CoA [86]. CoA serves as a precursor for the biosynthesis of acetate and butyrate [87]. Both acetate and butyrate metabolism have been shown to positively contribute to H_2_ production and are positively associated with increased CH4P [6]. Interestingly, *SWEET*, a sugar transporter found in eukaryotic organisms, suggests that higher abundances of fungi and protozoa may lead to increased CH4P [88, 89]. Additionally, the MG *hemD*, which plays a role in heme metabolism, has been reported to enhance CH4P during the anaerobic digestion of biomass waste [90]. The other MGs positively associated with CH4P show a range of functions that may support the overall development of the rumen microbiota, including methanogens. These functions involving several metabolic processes, such as peptide metabolism (*DPP3* [91], *map* [92]) and long chain fatty acid biosynthesis (*fabG* [93]) which can influence microbial growth, metabolism, physiology, and behaviour [94, 95]. Additionally, they play crucial roles in maintaining homeostasis, including cation balance (*KEA1_2_3* [96]) and cyclic-di-AMP homeostasis (*pgpH* [97]), which are essential for sustaining optimal internal conditions in microorganisms. Furthermore, these MGs are involved in the biosynthesis of microbial cell wall components, including *licD* [98], *bcrC* [99, 100], and *idi* [101].

Among the selected 43 MGs negatively associated with CH4P, we identified their involvement in a wide range of microbial activities that promote microbial resilience and sufficient nutrient metabolism, including amino acid metabolism (*serA*, *metA*, *sdaA*, *gltD* [102–105]), vitamin biosynthesis (*panE* [106]), nutrient transport (*pstS* [107]), and genetic information processes (DNA replication and repair: *dinJ*, *recD*, *recG*, *topB*, *umuC* [107–111]; ribosome biogenesis: *rsmB* [112]; and tRNA modifications: *tusA* [113]). Several MGs are also involved in microbial defence mechanisms, such as those related to the response against foreign DNA (*cas1* [114] and *cas2* [115]), and against environmental temperature stress, such as the cold shock protein: (*cspA* [116]) and the heat shock protein (*HSP20* [117], *hslO* [118]).

The flexibility of the multiple-trait model used for MDB allowed us to explore alternative strategies, such as addressing the unfavourable positive genetic correlation between ADG and CH4P. Our aim was to develop an MDB solution that reduces CH4P while avoiding negative impacts on ADG. Therefore, we identified 41 MGs that yielded favourable expected correlated responses in ADG and CH4P using the breeder equation, i.e., with positive responses for ADG and negative responses for CH4P. However, 14 of these MGs did not show strong probability of being favourably genetically correlated with both ADG and CH4P, as evidenced by their Pr0 values being smaller than 80% (but still > 60%), potentially due to our small sample size. Based on these MGs, we developed MDB41, which did not achieve the high accuracy and reduction in CH4P as obtained with MDB43, but still provided more than 70% of the reduction in CH4P while not compromising the response in ADG. In addition, MDB41 was similarly effective as the selection based on RCH4 in reducing CH4P. The MDB41 strategy did not result in a significant increase in ADG, most likely due to the substantially higher genetic variation in CH4P than in ADG in our population. By focusing on MG41 that have the genetic correlations r_gADG,MG_ with Pr0 > 80%, we identify different MGs but with functional characteristics similar to those of the MG43, which are negatively associated with CH4P. These include involvements in amino acid biosynthesis (*hisD* [119]), vitamin biosynthesis (*cbiD*, *cobP*, *cobT* [13, 120–122]), nucleotide metabolism (*ndk*, *pyrF*, *E2.7.6.5X* [123–125]), genetic information processing (*mfd*, *tfoS*, *dinB* [126–128]), compounds transporter (*amt*, *uup* [129, 130]) and sensing and responding to environmental changes (*ciaH*, *ettA* [131, 132]). In addition to these shared functions, the 41 MGs also support a well-functioning fermentation system, such as carbohydrate metabolism (*gmuG*, *kduI* [133, 134]), polysaccharide degradation (*afcA*, *abfA* [135, 136]), and starch degradation (*amyA* [137]). By consistently prioritizing the abundances of these MGs favourably associated with both CH4P and ADG, the MDB41 strategy offers a novel opportunity to directly enhance efficient functional pathways in the rumen to produce energy and protein, while reducing CH4 emissions through improved microbial growth acting as a hydrogen sink.

We demonstrated that MDB can be flexibly integrated into existing genetic evaluations, facilitated by multiple-trait model framework. By using abundances of MG43 as trait substitutes for CH4P in the multiple-trait model, together with DFI, ADG, and CCW, we achieved a high level of accuracy (0.81 ± 0.06) for CH4P and a selection response of −23.12% (95HPD: −28.07 to −16.98%, i = 1.76). These results exceeded those obtained based on a multiple-trait model that included production traits and directly measured CH4P. Higher accuracies using MDB were also obtained across production traits at 0.88 ± 0.07 vs. 0.83 ± 0.06 for DFI, 0.82 ± 0.09 vs. 0.73 ± 0.05 for ADG, and 0.92 ± 0.06 vs. 0.75 ± 0.05 for CCW. An even higher reduction in CH4P was achieved by incorporating direct measurements into the multiple-trait model alongside production traits and MG43. This combination resulted in the highest accuracy of 0.90 ± 0.09 and the largest expected selection response of −24.22% (95HPD: −27.27% to −21.37%) for CH4P per generation (with i = 1.76). However, it is important to highlight that this selection scenario resulted in a substantial estimated adverse response in ADG of -11.67% (95HPD: −14.79 to −8.48%) and thus is not a sustainable breeding strategy. In this case, we recommend the use of RCH4 as the selection criterion to avoid a reduction in ADG, which in our population also improved feed efficiency, as indicated by a reduced RFI.

A surprising result was the integration of MG41, which were favourably correlated with both CH4P reduction and ADG improvement, into the multiple-trait model with production traits. Selection based on GEBVs for CH4P, estimated solely from genetic correlations with both MG41 and production traits, resulted in a significant adverse correlated response in ADG of −10.79% (95HPD: −13.37 to −8.34%) and in a considerable desirable response in CH4P of −22.94% (95HPD: −25.15 to −20.69%). This is most likely due to the strong genetic correlations among DFI, ADG, and CCW, as well as between them and CH4P. These strong genetic correlations have more than negated the beneficial effects of MG41 on ADG so that the high reduction in CH4P is associated with a substantial negative response in ADG. Therefore, for strategies using MGs whose abundances are negatively associated with CH4P and positively associated with production traits, we recommend applying MDB41 in a separate genetic evaluation, rather than within a multiple-trait model with production traits, to predict GEBVs of CH4P. This strategy will not compromise ADG and promote efficient microbial pathways in the rumen, which desirably changing both traits.

The magnitude of the selection response for CH4P reported in this study should be interpreted in light of the fact that it was derived from a dataset collected on a single farm under controlled environmental conditions, with CH4P measured using gold-standard respiration chambers and considered as the sole selection objective. The reduction in selection response for CH4P obtained after imposing an additional aim of preventing adverse effects on ADG (e.g. using RCH4) already indicates the decline in genetic progress of CH4P, which likely to be greater when many traits are considered in the selection objective. Furthermore, the upper end of the range of selection intensities assumed in this study would only be achievable through intensive sire selection, in which case the expected genetic progress attributable to sires is only half of that presented in this study. Consequently, under practical production conditions, lower selection responses are expected due to simultaneous selection on multiple traits, greater environmental variation and differences in selection intensity between males and females. For the development of a selection index including CH4P or RCH4 alongside production traits, it should be recognised that the estimated total genetic merit is invariant to the choice of CH4 trait as similarly demonstrated by [23, 45] for feed efficiency.

Overall, the MDB strategy is very flexible, allowing the integration of production traits or the use of MGs favourably associated with those traits and CH4P. In particular, for reducing CH4P, MDB is expected to be a highly efficient strategy because it addresses the root cause of ruminal CH4P, the metabolism of excess hydrogen, which serves as an energy source for methanogenic archaea [5, 6, 138]. This is supported by the identification of MGs such as K03811, *rnfD*, and *oorD*, which are known to be directly or indirectly involved in H_2_ production [77, 80, 82, 83].

In this study, we used the most informative MGs within a multiple-trait model (MDB) to obtain GEBVs for CH4 emissions, rather than the methodology suggested by Difford et al. [139], which estimates a microbiome effect based on a microbial relationship matrix (**M**) and independently, an additive genetic effect using the genetic relationship matrix (**G**) (microbiability approach). We consider the latter approach conceptually problematic because CH4 is produced by rumen methanogenic archaea, whereas the host animal primarily influences the rumen environment rather than exerting a direct additive genetic effect on CH4 metabolism. Consequently, this approach does not adequately consider the genetic correlations between microbial activity and host genetics influencing the efficiency of ruminal microbial fermentation to reduce CH4 metabolisms and improve VFAs production and microbial protein synthesis for animal growth. In contrast, MDB centres on the most informative functionally relevant MGs that are under host genomic influence to predict the GEBVs of CH4 emissions. The multiple-trait model approach of MDB separately estimates the genetic and residual environmental (co)variances among abundances of the most informative MGs together with CH4 emissions and production traits. We acknowledge that fitting a multiple-trait model with numerous MGs treated as individual traits may increase the risk of overfitting. However, as we modelled only the most informative MGs, this risk was mitigated. The accuracy of the genetic and environmental parameters for MGs and CH4P requires further validation in independent datasets and may enable a further reduction in the number of MGs that need to be considered. Moreover, MDB strategy uses abundances of functionally relevant MGs rather than the overall microbial community. This strategy targets microbial pathways directly involved in ruminal fermentation and hydrogen metabolism of bacteria, fungi, and protozoa and is therefore expected to be more robust than approaches based on the rumen microbial taxa, which is often complex and incompletely classified, particularly for fungi and protozoa. In addition, the multiple-trait framework is flexible for identifying MGs that are favourably correlated with both CH4 mitigation and ADG. Selection based on the GEBVs of these MGs is therefore mitigating CH4 emissions without requiring direct CH4 measurements and without compromising growth, potentially across different breeds.

As with any new breeding system, the implementation of MDB will be challenging. First, rumen samples must be collected using a gastric tube, which takes on average five minutes per animal and requires skilled personnel. A key advantage is that a longitudinal ruminal sampling study has shown that a single sample per animal during the growing-finishing period is sufficient to accurately predict CH4 emissions and production traits [15]. Simpler sampling methods, such as saliva, which is easier to collect and shows promising associations with the rumen microbiome [140], are a likely next step for scaling up MDB applications. Microbial DNA extraction and sequencing is still costly; however, some costs could be reduced through targeted sequencing of the most informative MGs. In addition, a bioinformatic pipeline has to be developed to generate the abundances of the MGs from the sequence data. As the MDB strategy is based on a multiple-trait model, it can be implemented using existing genetic evaluation software. Routine genetic evaluation could be further simplified by using principal components of the abundances of the most informative MGs [141]. Furthermore, the microbiome composition can be used to simultaneously estimate the effects of the consumed diet (considering even selection of its components by individual animals) on CH4P. This trait appears to be more strongly affected by the diet composition than other traits such as feed efficiency and ADG [142]. They reported a 31% reduction in root mean square error for the genetic model when diet composition predicted by the microbiome profiles was additionally included in the genetic evaluations of CH4P. In addition, MDB can be simultaneously applied to improve feed efficiency [143, 144]; growth and its pattern [30], meat omeaga-3 fatty acid profiles [145] as well as to reduce ruminal pathogenic microbes [146] and potentially decrease ruminal antimicrobial resistance [147], all based on a single rumen sample without the need to directly measure these traits.

## Conclusions

Methane production in beef cattle was highly heritable and showed high expected selection responses, suggesting the success of animal breeding programmes targeting its reduction. To avoid the high recording costs of measuring CH4P, MDB was identified to be an efficient approach to change the metabolism of the rumen microbiota, which is the key to reduce CH4P. To further increase the accuracy of GEBVs for CH4P, the production traits such as DFI, ADG, and CCW can be easily integrated into MDB, as it is based on a multiple-trait model. However, the strong unfavourable correlations obtained in our population resulted in substantial reduction in ADG and CCW under selection for reduced CH4P. Therefore, we recommend the use of the developed selection criterion RCH4 that did not compromise production traits and even substantially improved feed efficiency.

Furthermore, the flexibility of the multiple-trait model used in MDB allows for the optimal combination of data with or without CH4P measurement to improve the accuracy of its GEBVs and can be easily extended to include additional novel phenotypes such as rumen bolus sensor information. Another promising application of MDB is the use of ruminal MGs with favourable genetic correlations to both CH4P and ADG, which promotes beneficial ruminal microbial functions and metabolism associated with both traits. This MDB strategy can reduce CH4P while avoiding adverse effects on production traits and is likely the most effective approach to directly select for more efficient functional pathways in the rumen. In general, this study offers applicable breeding strategies to effectively reduce CH4P from beef cattle without compromising productivity, thus providing a promising pathway towards more sustainable production of ruminants, with an emphasis on reducing their environmental impact.

## Supplementary Information

Below is the link to the electronic supplementary material.

Supplementary Material 1

Supplementary Material 2

## Data Availability

Metagenomic sequence reads for all rumen samples are available under European Nucleotide Archive (ENA) under accession projects PRJEB31266, PRJEB21624, PRJEB10338 and PRJEB70912.
