# Supplementary material for: Optimisation of selection for methane mitigation by integrating production traits with ruminal microbiome-driven breeding in beef cattle
Source: Genet Sel Evol. 2026 Jul 9;58:47. doi: 10.1186/s12711-026-01053-w (PMC13491708; doi:10.1186/s12711-026-01053-w)
Supplement: Supplementary file 1 [file 12711_2026_1053_MOESM2_ESM.docx]

**Table S1 Descriptive statistics of beef production traits and daily methane production**

| **Trait** | **N** | **Mean** | **SD** | **CV** |
| --- | --- | --- | --- | --- |
| Daily feed intake (kg/day) | 332 | 11.04 | 1.37 | 0.12 |
| Average daily gain (kg/day) | 352 | 1.476 | 0.281 | 0.19 |
| Feed conversion ratio (kg/kg) | 332 | 7.61 | 1.31 | 0.17 |
| Cold carcass weight (kg) | 359 | 372.9 | 37.1 | 0.10 |
| Daily methane production (g/day) | 287 | 190.5 | 52.8 | 0.28 |

N, Number of observations; SD, standard deviations; CV, coefficients of variation.

**Table S2 Expected selection responses based on selection for reduced daily methane production using genetic correlations estimated from the data**

| **Selection**  **intensity** | **Response** | **L.95HPD** | **H.95HPD** | **Pr0** | **Response**  **in percentage** | **L.95HPD in percentage** | **H.95HPD in percentage** |
| --- | --- | --- | --- | --- | --- | --- | --- |
| Daily feed intake (kg/day) | | | | |  |  |  |
| 2.06 | -1.42 | -1.75 | -1.03 | 1.00 | -12.82% | -15.83% | -9.31% |
| 1.76 | -1.18 | -1.40 | -0.95 | 1.00 | -10.70% | -12.65% | -8.58% |
| 1.40 | -0.97 | -1.10 | -0.85 | 1.00 | -8.75% | -9.96% | -7.72% |
| 1.16 | -0.80 | -0.90 | -0.71 | 1.00 | -7.23% | -8.12% | -6.46% |
| Residual feed intake (kg/day) | | | | |  |  |  |
| 2.06 | -0.26 | -0.52 | -0.05 | 0.99 | -3.45% | -6.82% | -0.64% |
| 1.76 | -0.24 | -0.40 | -0.08 | 1.00 | -3.14% | -5.22% | -1.05% |
| 1.40 | -0.16 | -0.26 | -0.07 | 1.00 | -2.11% | -3.38% | -0.90% |
| 1.16 | -0.14 | -0.21 | -0.06 | 1.00 | -1.78% | -2.81% | -0.84% |
| Average daily gain (kg/day) | | | | |  |  |  |
| 2.06 | -0.17 | -0.25 | -0.11 | 1.00 | -11.68% | -17.08% | -7.34% |
| 1.76 | -0.14 | -0.19 | -0.09 | 1.00 | -9.67% | -12.66% | -6.08% |
| 1.40 | -0.12 | -0.15 | -0.10 | 1.00 | -8.42% | -10.25% | -6.59% |
| 1.16 | -0.10 | -0.12 | -0.08 | 1.00 | -6.96% | -8.27% | -5.50% |
| Cold carcass weight (kg) | | | | |  |  |  |
| 2.06 | -23.28 | -31.80 | -15.70 | 1.00 | -6.24% | -8.53% | -4.21% |
| 1.76 | -18.38 | -24.29 | -13.86 | 1.00 | -4.93% | -6.51% | -3.72% |
| 1.40 | -14.85 | -17.94 | -12.21 | 1.00 | -3.98% | -4.81% | -3.27% |
| 1.16 | -12.09 | -14.17 | -10.07 | 1.00 | -3.24% | -3.80% | -2.70% |
| *Daily methane production (g/day)* | | | |  |  |  |  |
| 2.06 | -45.60 | -58.87 | -34.62 | 1.00 | -23.94% | -30.91% | -18.18% |
| 1.76 | -39.22 | -47.00 | -31.15 | 1.00 | -20.59% | -24.68% | -16.35% |
| 1.40 | -31.26 | -35.76 | -26.88 | 1.00 | -16.41% | -18.77% | -14.11% |
| 1.16 | -26.00 | -29.22 | -22.55 | 1.00 | -13.65% | -15.34% | -11.84% |
| Residual methane production (g/day) | | | | |  |  |  |
| 2.06 | -20.24 | -33.43 | -6.56 | 1.00 | -10.63% | -17.55% | -3.44% |
| 1.76 | -18.38 | -27.54 | -9.38 | 1.00 | -9.65% | -14.46% | -4.93% |
| 1.40 | -12.54 | -17.51 | -6.98 | 1.00 | -6.58% | -9.19% | -3.66% |
| 1.16 | -10.68 | -14.30 | -6.92 | 1.00 | -5.61% | -7.51% | -3.63% |

L.95HPD: lower limit of the 95% highest density interval, H.95HPD: upper limit of the 95% highest density interval. Pr0: probability of being positive or negative. Response in percentage of phenotypic mean of the traits.

**Table S3 Expected selection responses based on selection for reduced residual methane production using genetic correlations estimated from the data**

| **Selection**  **intensity** | **Response** | **L.95HPD** | **H.95HPD** | **Pr0** | **Response**  **in percentage** | **L.95HPD in percentage** | **H.95HPD in percentage** |
| --- | --- | --- | --- | --- | --- | --- | --- |
| Daily feed intake (kg/day) | | | | |  |  |  |
| 2.06 | -0.70 | -0.99 | -0.43 | 1.00 | -6.36% | -8.96% | -3.91% |
| 1.76 | -0.53 | -0.74 | -0.32 | 1.00 | -4.81% | -6.71% | -2.88% |
| 1.40 | -0.44 | -0.59 | -0.29 | 1.00 | -4.00% | -5.31% | -2.59% |
| 1.16 | -0.42 | -0.54 | -0.31 | 1.00 | -3.80% | -4.91% | -2.82% |
| Residual feed intake (kg/day) | | | | |  |  |  |
| 2.06 | -0.44 | -0.65 | -0.22 | 1.00 | -5.75% | -8.50% | -2.85% |
| 1.76 | -0.37 | -0.51 | -0.22 | 1.00 | -4.82% | -6.76% | -2.90% |
| 1.40 | -0.25 | -0.35 | -0.15 | 1.00 | -3.31% | -4.66% | -1.93% |
| 1.16 | -0.20 | -0.29 | -0.12 | 1.00 | -2.66% | -3.75% | -1.56% |
| Average daily gain (kg/day) | | | | |  |  |  |
| 2.06 | -0.04 | -0.10 | 0.03 | 0.87 | -2.38% | -6.93% | 1.70% |
| 1.76 | -0.02 | -0.06 | 0.03 | 0.77 | -1.06% | -4.12% | 2.02% |
| 1.40 | -0.02 | -0.06 | 0.00 | 0.94 | -1.55% | -3.74% | 0.32% |
| 1.16 | -0.03 | -0.05 | -0.01 | 0.99 | -1.87% | -3.46% | -0.36% |
| Cold carcass weight (kg) | | | | |  |  |  |
| 2.06 | -4.07 | -10.20 | 3.25 | 0.88 | -1.09% | -2.74% | 0.87% |
| 1.76 | -1.32 | -6.22 | 3.61 | 0.68 | -0.35% | -1.67% | 0.97% |
| 1.40 | -1.62 | -5.11 | 1.84 | 0.83 | -0.43% | -1.37% | 0.49% |
| 1.16 | -2.31 | -4.96 | 0.26 | 0.96 | -0.62% | -1.33% | 0.07% |
| Daily methane production (g/day) | | | |  |  |  |  |
| 2.06 | -34.83 | -47.32 | -23.38 | 1.00 | -18.29% | -24.84% | -12.28% |
| 1.76 | -27.69 | -36.80 | -19.53 | 1.00 | -14.54% | -19.32% | -10.25% |
| 1.40 | -22.39 | -27.92 | -16.05 | 1.00 | -11.75% | -14.66% | -8.43% |
| 1.16 | -19.34 | -24.46 | -15.01 | 1.00 | -10.15% | -12.84% | -7.88% |
| *Residual methane production (g/day)* | | | | |  |  |  |
| 2.06 | -30.27 | -41.18 | -19.79 | 1.00 | -15.89% | -21.62% | -10.39% |
| 1.76 | -26.05 | -33.66 | -17.69 | 1.00 | -13.68% | -17.67% | -9.29% |
| 1.40 | -20.22 | -25.97 | -15.18 | 1.00 | -10.62% | -13.63% | -7.97% |
| 1.16 | -16.44 | -20.90 | -12.16 | 1.00 | -8.63% | -10.97% | -6.38% |

L.95HPD: lower limit of the 95% highest density interval, H.95HPD: upper limit of the 95% highest density interval. Pr0: probability of being positive or negative. Response in percentage of phenotypic mean of the traits.

**Table S4 Expected selection responses based on selection for increased average daily gain using genetic correlations estimated from the data**

| **Selection**  **intensity** | **Response** | **L.95HPD** | **H.95HPD** | **Pr0** | **Response**  **in percentage** | **L.95HPD in percentage** | **H.95HPD in percentage** |
| --- | --- | --- | --- | --- | --- | --- | --- |
| Daily feed intake (kg/day) | | | | |  |  |  |
| 2.06 | 1.58 | 1.36 | 1.81 | 1.00 | 14.36% | 12.33% | 16.34% |
| 1.76 | 1.26 | 1.10 | 1.44 | 1.00 | 11.44% | 9.92% | 13.03% |
| 1.40 | 0.93 | 0.82 | 1.05 | 1.00 | 8.42% | 7.38% | 9.48% |
| 1.16 | 0.77 | 0.70 | 0.85 | 1.00 | 6.99% | 6.26% | 7.69% |
| Residual feed intake (kg/day) | | | | |  |  |  |
| 2.06 | 0.00 | -0.21 | 0.18 | 0.53 | -0.05% | -2.77% | 2.33% |
| 1.76 | -0.02 | -0.16 | 0.12 | 0.61 | -0.28% | -2.07% | 1.61% |
| 1.40 | -0.06 | -0.15 | 0.03 | 0.88 | -0.76% | -2.01% | 0.39% |
| 1.16 | -0.05 | -0.11 | 0.02 | 0.91 | -0.65% | -1.49% | 0.21% |
| *Average daily gain (kg/day)* | | | | |  |  |  |
| 2.06 | 0.25 | 0.20 | 0.31 | 1.00 | 17.27% | 13.84% | 21.04% |
| 1.76 | 0.21 | 0.17 | 0.25 | 1.00 | 14.11% | 11.39% | 16.80% |
| 1.40 | 0.16 | 0.14 | 0.19 | 1.00 | 10.95% | 9.20% | 12.89% |
| 1.16 | 0.13 | 0.12 | 0.15 | 1.00 | 9.09% | 7.91% | 10.45% |
| Cold carcass weight (kg) | | | | |  |  |  |
| 2.06 | 25.81 | 20.46 | 31.60 | 1.00 | 6.92% | 5.48% | 8.47% |
| 1.76 | 20.06 | 15.89 | 24.06 | 1.00 | 5.38% | 4.26% | 6.45% |
| 1.40 | 14.83 | 12.31 | 17.84 | 1.00 | 3.98% | 3.30% | 4.78% |
| 1.16 | 12.64 | 10.48 | 14.55 | 1.00 | 3.39% | 2.81% | 3.90% |
| Daily methane production (g/day) | | | |  |  |  |  |
| 2.06 | 43.68 | 35.75 | 52.22 | 1.00 | 22.93% | 18.77% | 27.42% |
| 1.76 | 34.85 | 28.66 | 40.56 | 1.00 | 18.30% | 15.05% | 21.30% |
| 1.40 | 24.55 | 20.71 | 28.70 | 1.00 | 12.89% | 10.87% | 15.07% |
| 1.16 | 20.24 | 17.23 | 23.26 | 1.00 | 10.62% | 9.05% | 12.21% |
| Residual methane production (g/day) | | | | |  |  |  |
| 2.06 | 3.72 | -6.79 | 16.01 | 0.71 | 1.95% | -3.57% | 8.40% |
| 1.76 | 5.36 | -2.07 | 14.07 | 0.90 | 2.81% | -1.09% | 7.39% |
| 1.40 | 5.09 | -0.16 | 11.37 | 0.96 | 2.67% | -0.08% | 5.97% |
| 1.16 | 2.34 | -1.84 | 6.44 | 0.86 | 1.23% | -0.97% | 3.38% |

L.95HPD: lower limit of the 95% highest density interval, H.95HPD: upper limit of the 95% highest density interval. Pr0: probability of being positive or negative. Response in percentage of phenotypic mean of the traits.

**Table S5 Expected selection responses based on selection for increased age-adjusted cold carcass weight using genetic correlations estimated from the data**

| **Selection**  **intensity** | **Response** | **L.95HPD** | **H.95HPD** | **Pr0** | **Response**  **in percentage** | **L.95HPD in percentage** | **H.95HPD in percentage** |
| --- | --- | --- | --- | --- | --- | --- | --- |
| Daily feed intake (kg/day) | | | | |  |  |  |
| 2.06 | 1.55 | 1.33 | 1.79 | 1.00 | 14.03% | 12.06% | 16.19% |
| 1.76 | 1.21 | 1.04 | 1.39 | 1.00 | 11.00% | 9.40% | 12.59% |
| 1.40 | 0.96 | 0.85 | 1.07 | 1.00 | 8.71% | 7.68% | 9.69% |
| 1.16 | 0.78 | 0.69 | 0.86 | 1.00 | 7.03% | 6.25% | 7.76% |
| Residual feed intake (kg/day) | | | | |  |  |  |
| 2.06 | 0.06 | -0.12 | 0.26 | 0.74 | 0.83% | -1.62% | 3.39% |
| 1.76 | 0.01 | -0.13 | 0.14 | 0.53 | 0.11% | -1.77% | 1.86% |
| 1.40 | 0.04 | -0.05 | 0.14 | 0.84 | 0.59% | -0.62% | 1.78% |
| 1.16 | 0.02 | -0.05 | 0.10 | 0.70 | 0.25% | -0.63% | 1.27% |
| Average daily gain (kg/day) | | | | |  |  |  |
| 2.06 | 0.23 | 0.17 | 0.28 | 1.00 | 15.36% | 11.38% | 18.91% |
| 1.76 | 0.19 | 0.15 | 0.23 | 1.00 | 12.55% | 9.88% | 15.31% |
| 1.40 | 0.14 | 0.11 | 0.17 | 1.00 | 9.53% | 7.66% | 11.18% |
| 1.16 | 0.12 | 0.10 | 0.14 | 1.00 | 7.87% | 6.46% | 9.17% |
| *Cold carcass weight (kg)* | | | | |  |  |  |
| 2.06 | 28.37 | 22.91 | 33.79 | 1.00 | 7.61% | 6.14% | 9.06% |
| 1.76 | 22.61 | 18.59 | 26.78 | 1.00 | 6.06% | 4.98% | 7.18% |
| 1.40 | 17.26 | 14.87 | 20.04 | 1.00 | 4.63% | 3.99% | 5.37% |
| 1.16 | 14.21 | 12.24 | 16.38 | 1.00 | 3.81% | 3.28% | 4.39% |
| Daily methane production (g/day) | | | |  |  |  |  |
| 2.06 | 42.44 | 33.83 | 50.64 | 1.00 | 22.28% | 17.76% | 26.59% |
| 1.76 | 32.13 | 26.15 | 38.82 | 1.00 | 16.87% | 13.73% | 20.38% |
| 1.40 | 26.71 | 22.89 | 30.72 | 1.00 | 14.03% | 12.02% | 16.13% |
| 1.16 | 20.98 | 17.71 | 23.79 | 1.00 | 11.01% | 9.30% | 12.49% |
| Residual methane production (g/day) | | | | |  |  |  |
| 2.06 | 3.26 | -8.41 | 15.48 | 0.71 | 1.71% | -4.41% | 8.13% |
| 1.76 | 0.81 | -7.24 | 8.43 | 0.57 | 0.43% | -3.80% | 4.43% |
| 1.40 | 2.30 | -3.56 | 7.51 | 0.80 | 1.21% | -1.87% | 3.94% |
| 1.16 | 1.57 | -2.91 | 5.83 | 0.76 | 0.82% | -1.53% | 3.06% |

L.95HPD: lower limit of the 95% highest density interval, H.95HPD: upper limit of the 95% highest density interval. Pr0: probability of being positive or negative. Response in percentage of phenotypic mean of the traits.

**Table S6 Expected selection responses based on selection for reduced residual feed intake using genetic correlations estimated from the data**

| **Selection**  **intensity** | **Response** | **L.95HPD** | **H.95HPD** | **Pr0** | **Response**  **in percentage** | **L.95HPD in percentage** | **H.95HPD in percentage** |
| --- | --- | --- | --- | --- | --- | --- | --- |
| Daily feed intake (kg/day) | | | | |  |  |  |
| 2.06 | -0.50 | -0.73 | -0.28 | 1.00 | -4.57% | -6.64% | -2.56% |
| 1.76 | -0.23 | -0.39 | -0.04 | 1.00 | -2.08% | -3.56% | -0.39% |
| 1.40 | -0.21 | -0.31 | -0.10 | 1.00 | -1.86% | -2.79% | -0.93% |
| 1.16 | -0.20 | -0.27 | -0.11 | 1.00 | -1.77% | -2.45% | -1.02% |
| *Residual feed intake (kg/day)* | | | | |  |  |  |
| 2.06 | -0.50 | -0.69 | -0.31 | 1.00 | -6.53% | -9.07% | -4.04% |
| 1.76 | -0.43 | -0.56 | -0.29 | 1.00 | -5.63% | -7.33% | -3.84% |
| 1.40 | -0.35 | -0.43 | -0.25 | 1.00 | -4.55% | -5.59% | -3.31% |
| 1.16 | -0.29 | -0.35 | -0.22 | 1.00 | -3.79% | -4.62% | -2.94% |
| Average daily gain (kg/day) | | | | |  |  |  |
| 2.06 | 0.01 | -0.05 | 0.06 | 0.64 | 0.66% | -3.31% | 4.28% |
| 1.76 | 0.04 | 0.00 | 0.08 | 0.98 | 2.84% | 0.04% | 5.37% |
| 1.40 | 0.03 | 0.00 | 0.05 | 0.99 | 2.01% | 0.25% | 3.71% |
| 1.16 | 0.02 | 0.00 | 0.04 | 0.99 | 1.47% | 0.15% | 2.68% |
| Cold carcass weight (kg) | | | | |  |  |  |
| 2.06 | -4.02 | -9.42 | 1.76 | 0.92 | -1.08% | -2.53% | 0.47% |
| 1.76 | -0.35 | -4.25 | 3.85 | 0.57 | -0.09% | -1.14% | 1.03% |
| 1.40 | -0.19 | -2.41 | 2.72 | 0.54 | -0.05% | -0.64% | 0.73% |
| 1.16 | -0.88 | -2.70 | 1.17 | 0.82 | -0.23% | -0.72% | 0.31% |
| Daily methane production (g/day) | | | |  |  |  |  |
| 2.06 | -28.69 | -37.18 | -21.42 | 1.00 | -15.06% | -19.52% | -11.25% |
| 1.76 | -18.87 | -25.14 | -13.36 | 1.00 | -9.91% | -13.20% | -7.01% |
| 1.40 | -16.00 | -19.64 | -12.25 | 1.00 | -8.40% | -10.31% | -6.43% |
| 1.16 | -13.79 | -16.91 | -11.04 | 1.00 | -7.24% | -8.88% | -5.80% |
| Residual methane production (g/day) | | | | |  |  |  |
| 2.06 | -25.63 | -36.60 | -17.02 | 1.00 | -13.46% | -19.22% | -8.94% |
| 1.76 | -19.55 | -27.48 | -13.29 | 1.00 | -10.26% | -14.43% | -6.98% |
| 1.40 | -15.10 | -20.14 | -10.57 | 1.00 | -7.93% | -10.57% | -5.55% |
| 1.16 | -12.28 | -15.87 | -8.61 | 1.00 | -6.45% | -8.33% | -4.52% |

L.95HPD: lower limit of the 95% highest density interval, H.95HPD: upper limit of the 95% highest density interval. Pr0: probability of being positive or negative. Response in percentage of phenotypic mean of the traits.

**Table S7 Expected selection responses based on selection for reduced daily methane production using low genetic correlations assumed to be equivalent to phenotypic correlations**

| **Selection**  **intensity** | **Response** | **L.95HPD** | **H.95HPD** | **Pr0** | **Response**  **in percentage** | **L.95HPD in percentage** | **H.95HPD in percentage** |
| --- | --- | --- | --- | --- | --- | --- | --- |
| Daily feed intake (kg/day) | | | | |  |  |  |
| 2.06 | -0.65 | -1.08 | -0.25 | 1.00 | -5.89% | -9.74% | -2.24% |
| 1.76 | -0.65 | -0.89 | -0.38 | 1.00 | -5.85% | -8.10% | -3.44% |
| 1.40 | -0.46 | -0.65 | -0.27 | 1.00 | -4.13% | -5.86% | -2.41% |
| 1.16 | -0.34 | -0.52 | -0.19 | 1.00 | -3.11% | -4.68% | -1.76% |
| Residual feed intake (kg/day) | | | | |  |  |  |
| 2.06 | -0.14 | -0.45 | 0.12 | 0.84 | -1.81% | -5.96% | 1.59% |
| 1.76 | -0.24 | -0.45 | -0.07 | 1.00 | -3.22% | -5.90% | -0.96% |
| 1.40 | -0.13 | -0.29 | 0.00 | 0.97 | -1.74% | -3.76% | 0.04% |
| 1.16 | -0.07 | -0.19 | 0.04 | 0.90 | -0.98% | -2.49% | 0.50% |
| Average daily gain (kg/day) | | | | |  |  |  |
| 2.06 | -0.05 | -0.12 | 0.02 | 0.89 | -3.05% | -7.94% | 1.30% |
| 1.76 | -0.04 | -0.09 | 0.01 | 0.96 | -2.95% | -5.89% | 0.48% |
| 1.40 | -0.04 | -0.08 | -0.01 | 0.99 | -2.96% | -5.11% | -0.46% |
| 1.16 | -0.04 | -0.06 | -0.01 | 1.00 | -2.39% | -4.27% | -0.46% |
| Cold carcass weight (kg/day) | | | | |  |  |  |
| 2.06 | -14.13 | -22.59 | -5.73 | 1.00 | -3.79% | -6.06% | -1.54% |
| 1.76 | -10.35 | -15.96 | -5.35 | 1.00 | -2.77% | -4.28% | -1.43% |
| 1.40 | -7.59 | -11.65 | -3.70 | 1.00 | -2.04% | -3.12% | -0.99% |
| 1.16 | -6.42 | -9.86 | -3.67 | 1.00 | -1.72% | -2.64% | -0.98% |
| *Daily methane production (g/day)* | | | |  |  |  |  |
| 2.06 | -40.06 | -53.99 | -26.90 | 1.00 | -21.03% | -28.35% | -14.13% |
| 1.76 | -33.91 | -43.39 | -25.49 | 1.00 | -17.80% | -22.78% | -13.38% |
| 1.40 | -26.98 | -34.20 | -20.35 | 1.00 | -14.16% | -17.96% | -10.69% |
| 1.16 | -22.08 | -27.72 | -16.89 | 1.00 | -11.59% | -14.56% | -8.87% |
| Residual methane production (g/day) | | | | |  |  |  |
| 2.06 | -27.22 | -41.35 | -12.89 | 1.00 | -14.29% | -21.71% | -6.77% |
| 1.76 | -24.10 | -32.93 | -14.86 | 1.00 | -12.65% | -17.29% | -7.80% |
| 1.40 | -19.32 | -26.39 | -13.05 | 1.00 | -10.14% | -13.86% | -6.85% |
| 1.16 | -15.67 | -21.20 | -10.60 | 1.00 | -8.22% | -11.13% | -5.56% |

L.95HPD: lower limit of the 95% highest density interval, H.95HPD: upper limit of the 95% highest density interval. Pr0: probability of being positive or negative. Response in percentage of phenotypic mean of the traits.

**Table S8 Expected selection responses based on selection for reduced residual methane production using low genetic correlations assumed to be equivalent to phenotypic correlations**

| **Selection**  **intensity** | **Response** | **L.95HPD** | **H.95HPD** | **Pr0** | **Response**  **in percentage** | **L.95HPD in percentage** | **H.95HPD in percentage** |
| --- | --- | --- | --- | --- | --- | --- | --- |
| Daily feed intake (kg/day) | | | | |  |  |  |
| 2.06 | -0.26 | -0.61 | 0.08 | 0.91 | -2.31% | -5.50% | 0.76% |
| 1.76 | -0.16 | -0.39 | 0.10 | 0.90 | -1.47% | -3.57% | 0.91% |
| 1.40 | -0.15 | -0.34 | 0.03 | 0.95 | -1.34% | -3.08% | 0.28% |
| 1.16 | -0.10 | -0.22 | 0.02 | 0.95 | -0.90% | -1.97% | 0.18% |
| Residual feed intake (kg/day) | | | | |  |  |  |
| 2.06 | -0.28 | -0.53 | -0.04 | 0.98 | -3.73% | -7.03% | -0.51% |
| 1.76 | -0.26 | -0.45 | -0.08 | 1.00 | -3.39% | -5.87% | -1.07% |
| 1.40 | -0.17 | -0.30 | -0.05 | 1.00 | -2.26% | -3.99% | -0.60% |
| 1.16 | -0.16 | -0.24 | -0.08 | 1.00 | -2.08% | -3.18% | -1.00% |
| Average daily gain (kg/day) | | | | |  |  |  |
| 2.06 | 0.02 | -0.05 | 0.07 | 0.69 | 1.09% | -3.36% | 5.06% |
| 1.76 | 0.02 | -0.02 | 0.07 | 0.84 | 1.58% | -1.17% | 4.59% |
| 1.40 | 0.00 | -0.03 | 0.03 | 0.53 | 0.05% | -2.16% | 2.16% |
| 1.16 | 0.01 | -0.02 | 0.03 | 0.72 | 0.43% | -1.02% | 1.87% |
| Cold carcass weight (kg) | | | | |  |  |  |
| 2.06 | -0.41 | -7.33 | 7.28 | 0.55 | -0.11% | -1.97% | 1.95% |
| 1.76 | 1.35 | -3.87 | 6.47 | 0.69 | 0.36% | -1.04% | 1.73% |
| 1.40 | 0.79 | -2.74 | 5.10 | 0.65 | 0.21% | -0.73% | 1.37% |
| 1.16 | 1.52 | -0.94 | 4.01 | 0.89 | 0.41% | -0.25% | 1.08% |
| Daily methane production (g/day) | | | |  |  |  |  |
| 2.06 | -34.13 | -46.47 | -22.04 | 1.00 | -17.92% | -24.40% | -11.57% |
| 1.76 | -27.89 | -36.04 | -19.35 | 1.00 | -14.64% | -18.92% | -10.16% |
| 1.40 | -22.58 | -28.52 | -16.11 | 1.00 | -11.85% | -14.97% | -8.46% |
| 1.16 | -17.65 | -21.60 | -13.28 | 1.00 | -9.27% | -11.34% | -6.97% |
| *Residual methane production (g/day)* | | | | |  |  |  |
| 2.06 | -34.44 | -45.89 | -22.07 | 1.00 | -18.08% | -24.09% | -11.59% |
| 1.76 | -29.85 | -38.79 | -22.42 | 1.00 | -15.67% | -20.36% | -11.77% |
| 1.40 | -23.22 | -30.21 | -17.34 | 1.00 | -12.19% | -15.86% | -9.11% |
| 1.16 | -19.09 | -23.21 | -14.88 | 1.00 | -10.02% | -12.19% | -7.81% |

L.95HPD: lower limit of the 95% highest density interval, H.95HPD: upper limit of the 95% highest density interval. Pr0: probability of being positive or negative. Response in percentage of phenotypic mean of the traits.

**Table S9 Expected selection responses based on selection for increased average daily gain using low genetic correlations assumed to be equivalent to phenotypic correlations**

| **Selection**  **intensity** | **Response** | **L.95HPD** | **H.95HPD** | **Pr0** | **Response**  **in percentage** | **L.95HPD in percentage** | **H.95HPD in percentage** |
| --- | --- | --- | --- | --- | --- | --- | --- |
| Daily feed intake (kg/day) | | | | |  |  |  |
| 2.06 | 1.10 | 0.82 | 1.36 | 1.00 | 9.93% | 7.43% | 12.32% |
| 1.76 | 0.78 | 0.59 | 0.99 | 1.00 | 7.08% | 5.31% | 8.95% |
| 1.40 | 0.57 | 0.41 | 0.71 | 1.00 | 5.12% | 3.73% | 6.41% |
| 1.16 | 0.46 | 0.35 | 0.56 | 1.00 | 4.17% | 3.20% | 5.03% |
| Residual feed intake (kg/day) | | | | |  |  |  |
| 2.06 | 0.22 | 0.03 | 0.40 | 0.99 | 2.86% | 0.41% | 5.29% |
| 1.76 | 0.13 | -0.02 | 0.28 | 0.95 | 1.70% | -0.23% | 3.71% |
| 1.40 | 0.06 | -0.05 | 0.17 | 0.86 | 0.80% | -0.59% | 2.28% |
| 1.16 | 0.05 | -0.02 | 0.13 | 0.91 | 0.70% | -0.25% | 1.75% |
| *Average daily gain (kg/day)* | | | | |  |  |  |
| 2.06 | 0.19 | 0.14 | 0.24 | 1.00 | 12.65% | 9.41% | 16.39% |
| 1.76 | 0.16 | 0.12 | 0.20 | 1.00 | 10.73% | 8.04% | 13.37% |
| 1.40 | 0.13 | 0.10 | 0.15 | 1.00 | 8.48% | 6.52% | 10.25% |
| 1.16 | 0.10 | 0.08 | 0.12 | 1.00 | 6.94% | 5.61% | 8.28% |
| Cold carcass weight (kg) | | | | |  |  |  |
| 2.06 | 14.67 | 8.64 | 20.55 | 1.00 | 3.93% | 2.32% | 5.51% |
| 1.76 | 9.16 | 5.35 | 13.63 | 1.00 | 2.46% | 1.43% | 3.65% |
| 1.40 | 6.88 | 3.83 | 10.09 | 1.00 | 1.84% | 1.03% | 2.71% |
| 1.16 | 5.41 | 3.31 | 7.77 | 1.00 | 1.45% | 0.89% | 2.08% |
| Daily methane production (g/day) | | | |  |  |  |  |
| 2.06 | 10.61 | 1.22 | 20.49 | 0.99 | 5.57% | 0.64% | 10.76% |
| 1.76 | 11.60 | 4.76 | 19.08 | 1.00 | 6.09% | 2.50% | 10.02% |
| 1.40 | 11.86 | 6.49 | 17.67 | 1.00 | 6.23% | 3.41% | 9.28% |
| 1.16 | 8.04 | 3.87 | 11.80 | 1.00 | 4.22% | 2.03% | 6.20% |
| Residual methane production (g/day) | | | | |  |  |  |
| 2.06 | -8.19 | -18.00 | 0.82 | 0.95 | -4.30% | -9.45% | 0.43% |
| 1.76 | -1.77 | -8.69 | 6.07 | 0.67 | -0.93% | -4.56% | 3.19% |
| 1.40 | 1.58 | -3.60 | 6.98 | 0.71 | 0.83% | -1.89% | 3.67% |
| 1.16 | -0.20 | -4.16 | 3.59 | 0.54 | -0.11% | -2.18% | 1.89% |

L.95HPD: lower limit of the 95% highest density interval, H.95HPD: upper limit of the 95% highest density interval. Pr0: probability of being positive or negative. Response in percentage of phenotypic mean of the traits.

**Table S10 Expected selection responses based on selection for increased age-adjusted cold carcass weight using low genetic correlations assumed to be equivalent to phenotypic correlations**

| **Selection**  **intensity** | **Response** | **L.95HPD** | **H.95HPD** | **Pr0** | **Response**  **in percentage** | **L.95HPD in percentage** | **H.95HPD in percentage** |
| --- | --- | --- | --- | --- | --- | --- | --- |
| Daily feed intake (kg/day) | | | | |  |  |  |
| 2.06 | 0.99 | 0.67 | 1.29 | 1.00 | 9.00% | 6.05% | 11.65% |
| 1.76 | 0.79 | 0.59 | 1.02 | 1.00 | 7.19% | 5.35% | 9.20% |
| 1.40 | 0.60 | 0.46 | 0.73 | 1.00 | 5.41% | 4.18% | 6.58% |
| 1.16 | 0.51 | 0.41 | 0.61 | 1.00 | 4.64% | 3.74% | 5.52% |
| Residual feed intake (kg/day) | | | | |  |  |  |
| 2.06 | 0.07 | -0.13 | 0.31 | 0.73 | 0.97% | -1.68% | 4.05% |
| 1.76 | 0.02 | -0.13 | 0.16 | 0.64 | 0.28% | -1.73% | 2.14% |
| 1.40 | 0.02 | -0.08 | 0.11 | 0.63 | 0.21% | -1.01% | 1.45% |
| 1.16 | 0.04 | -0.03 | 0.12 | 0.86 | 0.52% | -0.38% | 1.52% |
| Average daily gain (kg/day) | | | | |  |  |  |
| 2.06 | 0.09 | 0.02 | 0.14 | 1.00 | 5.77% | 1.32% | 9.77% |
| 1.76 | 0.07 | 0.03 | 0.11 | 1.00 | 5.08% | 2.00% | 7.69% |
| 1.40 | 0.05 | 0.03 | 0.08 | 1.00 | 3.59% | 1.88% | 5.39% |
| 1.16 | 0.04 | 0.02 | 0.06 | 1.00 | 2.84% | 1.55% | 4.38% |
| *Cold carcass weight (kg)* | | | | |  |  |  |
| 2.06 | 25.03 | 18.75 | 31.40 | 1.00 | 6.71% | 5.03% | 8.42% |
| 1.76 | 20.71 | 16.62 | 25.77 | 1.00 | 5.55% | 4.46% | 6.91% |
| 1.40 | 15.86 | 13.15 | 18.73 | 1.00 | 4.25% | 3.53% | 5.02% |
| 1.16 | 13.02 | 10.78 | 15.18 | 1.00 | 3.49% | 2.89% | 4.07% |
| Daily methane production (g/day) | | | |  |  |  |  |
| 2.06 | 19.38 | 7.29 | 30.74 | 1.00 | 10.17% | 3.83% | 16.14% |
| 1.76 | 15.43 | 7.19 | 23.47 | 1.00 | 8.10% | 3.77% | 12.32% |
| 1.40 | 10.25 | 5.55 | 15.24 | 1.00 | 5.38% | 2.91% | 8.00% |
| 1.16 | 8.98 | 4.96 | 12.96 | 1.00 | 4.71% | 2.61% | 6.81% |
| Residual methane production (g/day) | | | | |  |  |  |
| 2.06 | -3.57 | -14.97 | 8.09 | 0.73 | -1.88% | -7.86% | 4.25% |
| 1.76 | -3.74 | -11.78 | 3.92 | 0.81 | -1.96% | -6.19% | 2.06% |
| 1.40 | -4.26 | -9.10 | 0.52 | 0.96 | -2.24% | -4.78% | 0.27% |
| 1.16 | -2.86 | -6.48 | 1.04 | 0.92 | -1.50% | -3.40% | 0.55% |

L.95HPD: lower limit of the 95% highest density interval, H.95HPD: upper limit of the 95% highest density interval. Pr0: probability of being positive or negative. Response in percentage of phenotypic mean of the traits.

**Table S11 Expected selection responses based on selection for reduced residual feed intake using low genetic correlations assumed to be equivalent to phenotypic correlations**

| **Selection**  **intensity** | **Response** | **L.95HPD** | **H.95HPD** | **Pr0** | **Response**  **in percentage** | **L.95HPD in percentage** | **H.95HPD in percentage** |
| --- | --- | --- | --- | --- | --- | --- | --- |
| Daily feed intake (kg/day) | | | | |  |  |  |
| 2.06 | -0.75 | -0.98 | -0.47 | 1.00 | -6.76% | -8.91% | -4.27% |
| 1.76 | -0.67 | -0.85 | -0.43 | 1.00 | -6.04% | -7.70% | -3.93% |
| 1.40 | -0.60 | -0.72 | -0.44 | 1.00 | -5.41% | -6.55% | -3.99% |
| 1.16 | -0.50 | -0.61 | -0.38 | 1.00 | -4.51% | -5.53% | -3.49% |
| *Residual feed intake (kg/day)* | | | | |  |  |  |
| 2.06 | -0.87 | -1.08 | -0.69 | 1.00 | -11.49% | -14.14% | -9.02% |
| 1.76 | -0.75 | -0.89 | -0.60 | 1.00 | -9.80% | -11.75% | -7.95% |
| 1.40 | -0.60 | -0.70 | -0.49 | 1.00 | -7.84% | -9.18% | -6.49% |
| 1.16 | -0.49 | -0.57 | -0.41 | 1.00 | -6.43% | -7.44% | -5.33% |
| Average daily gain (kg/day) | | | | |  |  |  |
| 2.06 | 0.01 | -0.04 | 0.07 | 0.66 | 0.79% | -2.41% | 4.46% |
| 1.76 | -0.01 | -0.05 | 0.03 | 0.58 | -0.34% | -3.08% | 2.34% |
| 1.40 | -0.02 | -0.04 | 0.02 | 0.86 | -1.02% | -2.77% | 1.02% |
| 1.16 | -0.01 | -0.03 | 0.01 | 0.88 | -0.94% | -2.34% | 0.54% |
| Cold carcass weight (kg) | | | | |  |  |  |
| 2.06 | 3.49 | -2.50 | 8.83 | 0.88 | 0.94% | -0.67% | 2.37% |
| 1.76 | 3.20 | -0.67 | 8.54 | 0.92 | 0.86% | -0.18% | 2.29% |
| 1.40 | 1.31 | -1.61 | 4.12 | 0.82 | 0.35% | -0.43% | 1.10% |
| 1.16 | 0.90 | -1.32 | 3.13 | 0.77 | 0.24% | -0.35% | 0.84% |
| Daily methane production (g/day) | | | |  |  |  |  |
| 2.06 | -4.37 | -13.48 | 6.19 | 0.80 | -2.29% | -7.08% | 3.25% |
| 1.76 | -6.79 | -14.11 | 0.00 | 0.97 | -3.56% | -7.41% | 0.00% |
| 1.40 | -5.70 | -10.15 | -1.04 | 0.99 | -2.99% | -5.33% | -0.55% |
| 1.16 | -6.45 | -10.09 | -2.73 | 1.00 | -3.39% | -5.30% | -1.43% |
| Residual methane production (g/day) | | | | |  |  |  |
| 2.06 | -4.55 | -14.51 | 5.47 | 0.81 | -2.39% | -7.62% | 2.87% |
| 1.76 | -9.05 | -16.35 | -2.11 | 0.99 | -4.75% | -8.58% | -1.11% |
| 1.40 | -6.39 | -11.33 | -1.99 | 1.00 | -3.36% | -5.95% | -1.04% |
| 1.16 | -6.59 | -9.80 | -2.85 | 1.00 | -3.46% | -5.14% | -1.50% |

L.95HPD: lower limit of the 95% highest density interval, H L.95HPD: upper limit of the 95% highest density interval. Pr0: probability of being positive or negative. Response in percentage of phenotypic mean of the traits.
